# GWAS hints at pleiotropic roles for *FLOWERING LOCUS T* in flowering time and yield-related traits in canola

**DOI:** 10.1186/s12864-019-5964-y

**Published:** 2019-08-06

**Authors:** Harsh Raman, Rosy Raman, Yu Qiu, Avilash Singh Yadav, Sridevi Sureshkumar, Lauren Borg, Maheswaran Rohan, David Wheeler, Oliver Owen, Ian Menz, Sureshkumar Balasubramanian

**Affiliations:** 1 NSW Department of Primary Industries, Wagga Wagga Agricultural Institute, Wagga Wagga, NSW 2650 Australia; 2 School of Biological Sciences, Monash University, Clayton, VIC3800 Australia; 3 Centre for Bioinformatics and Biometrics, University of Wollongong, Wollongong, NSW 2522 Australia; 4 NSW Department of Primary Industries, Orange Agricultural Institute, Orange, NSW 2800 Australia

**Keywords:** Natural variation, Flowering time, Canola, Photoperiod, genome-wide association analysis, linkage analysis, Gene expression, eQTL analysis

## Abstract

**Background:**

Transition to flowering at the right time is critical for local adaptation and to maximize grain yield in crops. Canola is an important oilseed crop with extensive variation in flowering time among varieties. However, our understanding of underlying genes and their role in canola productivity is limited.

**Results:**

We report our analyses of a diverse GWAS panel (300–368 accessions) of canola and identify SNPs that are significantly associated with variation in flowering time and response to photoperiod across multiple locations. We show that several of these associations map in the vicinity of *FLOWERING LOCUS T* (*FT*) paralogs and its known transcriptional regulators. Complementary QTL and eQTL mapping studies, conducted in an Australian doubled haploid population, also detected consistent genomic regions close to the *FT* paralogs associated with flowering time and yield-related traits. *FT* sequences vary between accessions. Expression levels of *FT* in plants grown in field (or under controlled environment cabinets) correlated with flowering time. We show that markers linked to the *FT* paralogs display association with variation in multiple traits including flowering time, plant emergence, shoot biomass and grain yield.

**Conclusions:**

Our findings suggest that *FT* paralogs not only control flowering time but also modulate yield-related productivity traits in canola.

**Electronic supplementary material:**

The online version of this article (10.1186/s12864-019-5964-y) contains supplementary material, which is available to authorized users.

## Highlight

The genetic association, eQTL and expression analyses suggest that *FT* paralogs have multifaceted roles in canola flowering time, plant development and productivity traits.

## One sentence summary

Paralogs of *FT* which is known to be critical for flowering time have pleiotropic roles in yield related traits in canola.

## Background

Natural variation provides a valuable resource for discovering the genetic and molecular basis of phenotypic diversity in plant development, adaptation and productivity [1, 2]. Canola (rapeseed, *Brassica napus* L., A_n_A_n_C_n_C_n_ genomes, 2n = 4× =38) is an important oil crop, varieties of which display extensive variation in life history traits such as flowering time. Precise knowledge of flowering time is fundamental for identifying locally adapted varieties. It is also essential in the development of new varieties that maximize yield and oil quality in diverse and rapidly changing environments. For example, early flowering varieties are preferred for cultivation when periods of drought and high heat are frequent, whereas winter/semi-winter crops achieve maximum yields in the longer growing seasons that occur in temperate regions [3].

In *Arabidopsis thaliana*, the four major pathways that regulate flowering time are photoperiod, vernalisation, autonomous and gibberellic acid pathways [4, 5]. MicroRNAs, sugar status and signaling also interact with the flowering pathways to generate a complex regulatory network [6]. Flowering is also affected by other external factors such as ambient temperature, insect-pests, pathogens, light quality, and abiotic stress [1, 7]. Genetic analyses based on classical linkage mapping (quantitative trait loci: QTLs) and genome-wide association studies (GWAS) have revealed that flowering time in canola is a multigenic trait [8–16]. Candidate genes underlying flowering time variation due to vernalisation have been identified in *B. napus* [8, 12, 17–21]. We have previously shown that *BnFLC.A02* accounts for the majority (~ 23%) of variation in flowering time among diverse accessions of canola [12]. Nevertheless, little is known about functional role of the photoperiod responsive genes in modulating flowering time especially in spring canola varieties.

The gene *FLOWERING LOCUS T* (*FT*) is generally considered to integrate inputs from several pathways that finally result in floral transition. In *A. thaliana,* loss-of-function mutations in *FT* result in late flowering under long-day conditions [22, 23]. In *B. napus*, six paralogs of *FT* have been identified [24, 25] that contribute to functional divergence in flowering time between winter and spring cultivars. For example, mutations in *BnC6.FTa and BnC6.FTb* paralogs have been shown to alter flowering time in *B. napus* accessions [26]. Owing to the multiple copies of *FT* in canola, it has been difficult to establish the functionality and precise relationship between various paralogs in plant development and productivity traits, as shown in Arabidopsis, onion and potato [27–32]. In addition, under field conditions, it is difficult to determine the extent of genetic variation in photoperiod response, as plants undergo a series of cold temperature-episodes during vernalisation.

Here we determine the extent of flowering time variation utilizing a diverse panel of 368 canola genotypes representing different geographic locations around the world. Using GWAS we identify several underlying QTLs controlling phenotypic variation in photoperiod response and flowering time. We show that the response to photoperiod maps to *FT* paralogues, and their potential transcriptional regulators *CIB*, *CO*, *CRY2*, *FVE*, *MSI*, *EMF2* and *PIF4*. Using a doubled haploid population of plants grown under LD and/or field conditions, we show that expression levels of *FT* paralogs are significantly associated with flowering time variation across diverse canola accessions. The eQTL analysis for *FT* expression levels map not only to *FT* itself (e.g., *BnA7.FT*) but also other loci that are known regulators of *FT* such as *BnFLC.C3b* (*FLC5*), *FPA*, *SPA1* and *ELF4*. We also demonstrate that plant productivity traits such as plant emergence, shoot biomass accumulation, plant height, and grain yield map in the vicinity of *FT*. Taken together our findings suggest that *FT* has multifaceted role in plants and could be exploited for selection of canola varieties for improved productivity.

## Materials and methods

### Plant material and growth conditions

#### Evaluation of GWAS panel

A diverse panel of 368 accessions of *B. napus* L. was used to evaluate photoperiod response in this study (Additional file 1: Table S1). A 300 accessions subset of these was evaluated for flowering time in three field experiments: (a) in plots (35°03′36.9″S 147°18′40.2″E, 147 m above sea level) at the Wagga Wagga Agricultural Institute (WWAI) located at Wagga Wagga, NSW, Australia, (b) in plots at the Condobolin Agricultural Research and Advisory Station, NSW, Australia (33. 0418.98°S, 147.1350.16°E, 220 m above sea level) and (c) in single rows at WWAI (35°02′27.0″S 147°19′12.6″E) in 2017 canola growing season. For WWAI plot trial, 300 accessions were arranged in a randomized complete block design with 60 rows by 10 columns (ranges) in four flood irrigation bays, each bay had 15 rows and 10 ranges (Additional file 2: Table S2). A buffer (non-experimental line) row of an Australian canola variety SturtTT was seeded after every two ranges to ensure that plots are harvested at the right maturity. For WWAI single row trial, 300 accessions were arranged in a randomized block design with 60 rows (each row 10 M long) by 10 columns in two replicates (Additional file 2: Table S2), each replicate of 30 rows was separated with a buffer row of SturtTT. Each accession per replication had 100 plants. This trial was sown under Lateral Move irrigation system to match water demand for optimal plant growth. The Condobolin trial was sown as rainfed and arranged in a randomised complete block design with 100 rows by 6 columns, accommodating all 300 accessions in two replicates (Additional file 2: Table S2). For field plot experiments, accessions were sown in plots (2 m wide × 10 m long at Wagga Wagga and 2 m wide × 12 m long at Condobolin) at density of 1400 seeds/20 m^2^ plot. Seeds were counted with Kimseed machine and directly sown in plots in the field; each plot consisted of 6 rows spaced 25 cm apart. Plots were sown with a six-row cone-seeder to 10 m length. All plots were sown with a granular fertilizer (N: P: K: S, 22: 1: 0: 15) applied at 150 kg ha^–P^. The fertilizer was treated with the fungicide Jubilee (a.i. flutriafol at 250 g/L, Farmoz Pty Ltd., St Leonards, NSW, Australia) to protect all genotypes against the blackleg fungus, *Leptosphaeria maculans*. After crop establishment, plots were trimmed back to 8 m after emergence by applying Roundup (a. i. glyphosate) herbicide with a shielded spray boom. For controlled environmental cabinets (CE cabinets, Thermoline Scientific, Wetherill Park NSW, Australia), eight plants of each of the 368 accessions were grown in plastic trays as described previously [12] under long (LD) and short day (SD) conditions. For LD treatment, seeds were planted in a CE maintained at 20 ± 1 °C under white fluorescent lamps (4000 K, Osram) with light intensity of approximately 150 μM/m^2^/s, with a 16-h photoperiod. In SD treatment, plants from 368 accessions were grown at the same conditions described above but for 8 h photoperiod.

#### Flowering time and other phenotypic measurements

**Fig. 1:**
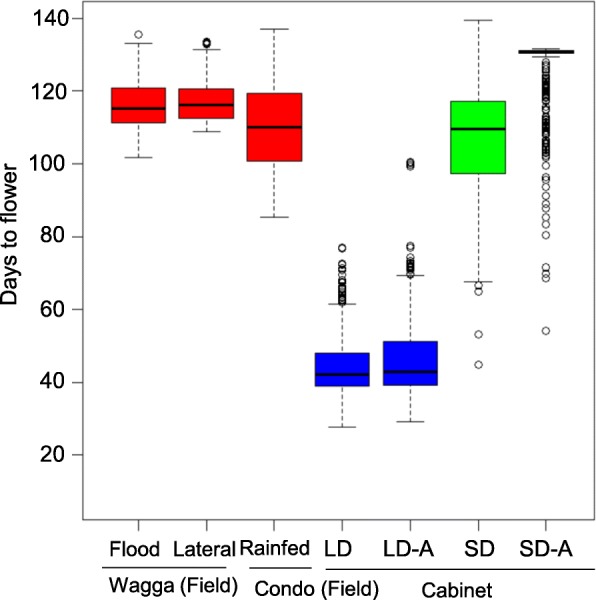
Natural variation for flowering time. Box-plots showing genetic variation for flowering time in a diverse panel of canola accessions, which were grown across five experiments under field, and controlled environment conditions (cabinets). Days to flowering were scored in 2017: Field plots at Wagga Wagga (flood irrigated) and Condobolin (Condo, rainfed); Single rows at Wagga Wagga (lateral move irrigation); Days to flowering were scored in long day condition (LD, 16 h) and short day condition (SD, 8 h) plants under cabinets. Genotypes that did not flower till the end of experiment were also included and marked as flowering ‘assigned’ (LD-A and SD-A). A total of 368 accessions were evaluated for flowering time under LD and SD conditions, while 300 accessions were evaluated under field conditions. Details are given in Additional file 1: Table S1

Days to flower from sowing was calculated when 50% of plants had opened their first flower. In SD conditions, flowering time was recorded for up to 200 days. Plants without any flowers at the end of the experiments were classified as ‘assigned (A)’ (LD-A, SD-A, see Fig. 1). The response to photoperiod was calculated as the difference between 50% flowering in plants grown under SD and LD conditions. For field trials, flowering time was recorded three times in a week.

Normalised Difference Vegetative Index (NDVI) was measured as a proxy of fractional ground cover for early vigour [33, 34] using a GreenSeeker® (model 505, NTech Industries Inc., Ukiah, CA, USA). The NDVI readings were taken at 7–10 days interval after 5 weeks of sowing before the onset of flowering. Multiple readings were taken in each plot and then averaged across each plot for genetic analysis. Plots were harvested by direct heading with a Kingroy plot harvester (Kingaroy Engineering Works, Queensland, Australia) in the 4th wk. of November (Condobolin, NSW) and 2–3rd wk. of December (Wagga, Australia). Grain samples were cleaned with Kimseed (Kimseed Australia, Western Australia) and plot yield was expressed into t/ha.

#### Field evaluation of SAgS DH population

SAgS population of 144 DH progeny from a BC_1_F_1_ plant derived from the cross Skipton (less responsive to vernalisation) and Ag-Spectrum (more responsive to vernalisation) have been previously described [12, 13, 35]). The population was grown in 2015 (35°01′32.3″S 147°19′25.4″E) and 2016 (35°01′42.8″S, 147°20′23.3″E) in the field at the WWAI, NSW, Australia. Both trials were randomised in a complete block design with three replicates in a single block. A total of 1,400 seeds per genotype were directly sown in plots in the field as described above. The traits measured included plant emergence, first flowering, plant biomass, plant height, and grain yield. Plant (shoot) biomass was calculated from cuttings obtained from 10 randomly selected plants growing in the central row of each plot. Each sample was weighed on a digital scale and fresh weights were expressed in g/plant. Plant height (cm) was measured at the physiological maturity stage using 5 plants selected randomly in the middle row of each plot. Plots were harvested with a Kingaroy plot harvester in the 2–3rd wk. of December (Wagga, Australia).

#### Genome-wide genotyping

Leaf material was collected individually from 368 diverse DH canola accessions, grown under LD conditions, and then immediately snap-frozen in liquid nitrogen. Genomic DNA was isolated as described previously [13] and sent to Trait Genetics, Germany (http://www.traitgenetics.com/) for genotyping with Illumina infinium 15 k *Brassica* chip representing 60 K Infinium SNP array [36].

### Population structure and GWA analyses

For GWA analysis, we only used SNP markers with allele frequencies > 0.05 and overall call rates (proportion of genotypes per marker) of > 90% [37]. To prevent the potential loss of genome wide associations (GWA) missing data was imputed [38]. A total of 11,804 SNP markers could be anchored to the A_n_ and C_n_ subgenomes of reference sequenced genome of *B. napus cv.* The variety *‘*Darmor-*bzh’* (Darmor) was used as reference for cluster and GWA analyses in a diversity panel of 368 accessions (Additional file 1: Table S1). Cluster analysis was performed with the Neighbor-Joining method [39] using MEGA version 6. In order to reduce spurious associations between markers and variation in flowering time, population structure and the relative kinship coefficients of individual genotypes were estimated as described previously [12]. Flowering time-SNP marker association analysis was performed using the EMMAx/P3D method [40, 41] implemented in the R package GAPIT [42] (https://cran.r-project.org/). Significance of GWA between markers and flowering time was tested at LOD score of 3. The *P* (−log_10_*P*) values for each SNP were exported to generate a Manhattan plot in R [43]. The proximity of candidate genes to identified associations based on the physical positions of SNPs/candidate genes was inferred based on functional annotation of the *A. thaliana* genome and implemented in the reference sequenced genome of Darmor [44]. After Bonferroni correction, associations with LOD score = 5.41 were also considered as significant on a *p* < 0.05 level. The associations detected through GWAS were compared with the QTL intervals associated with flowering time identified in the field conditions in the SAgS DH mapping population evaluated in 2013, 2014 [13], 2015 and 2016 (this study).

### Statistical and QTL analysis

Flowering and other phenotypic data collected from different experiments were analysed using linear mixed models in R as described previously [45]. Essentially, we defined the individual experimental Plot as a factor with 432 levels for each of the 2015 and 2016 trials. The factors: Row and Range corresponded to the rows and ranges of the trials, with levels equal to the number of rows and ranges in each trial. The combination of levels of Row and Range completely index the levels of Plot such that Plot = Row:Range. The factor Rep has 3 levels corresponding to the replicate blocks in each trial. The plot structure for the field experiment consists of plots nested within blocks and is given by, Rep/Plot which can be expanded to give, Rep + Rep:Plot. The term Rep:Plot indexes the observational units for all traits and thus is equivalent to the residual term for these traits. The treatments for the field phase of the experiment are the lines allocated to plots so we define the treatment factor, Genotype, with 144 levels corresponding to lines grown in each trial. Due to marker data being included in the model, we need to define an additional two factors; Gkeep (corresponding to lines with both phenotypic and marker data) and Gdrop. The factor Gdrop has 16 levels corresponding to lines with phenotypic data but not marker data. Therefore treatment structure is given by Gkeep + Gdrop. Finally, marker data is incorporated into the analysis and individual markers are scanned following the approach of Nelson et al. (2014) [9] to establish a final multi-QTL model. We also used phenotypic data from 2013 and 2014 experiments that was published previously [13], in order to test multifaceted role of *FT* in flowering time and other productivity traits across environments. A genetic map based on 7,716 DArTseq markers representing 499 unique loci [13] was used to determine trait-marker associations. The predicted means for first flowering, and response to photoperiod for each genotype were used to detect genome wide trait-marker associations.

### *FT* expression and eQTL analyses

*FT* expression analysis was carried out in two different sets of populations. First, we analysed *FT* expression in field-grown plants from 144 DH lines of the SAgS DH population. Second, we analysed *FT* expression in 24 accessions that represented extreme flowering phenotypes (i.e., early and late flowering accessions) from the 368 accessions in the GWAS panel. For both sets of experiments, five independent leaf samples collected from field/CE grown plants (at floral budding stage) per genotype were pooled and flash-frozen in liquid nitrogen (in field/CE). For field-grown plants there were internal replications that effectively represented at least two biological replicates. For CE grown plants three biological replicates were used. RNA was isolated using TRIZol (Invitrogen) and cDNA was synthesized using a First Strand Synthesis Kit (Roche). Samples were controlled for their quality using two approaches as outlined previously [12]. Gene specific primers for each of six *FT* paralogs [26] used for the expression analysis are described in Additional file 3: Table S3. Since the expression levels of all *FT* paralogs were correlated, we used data from *BnC6.FT* for eQTL analysis using SVS package (Golden Helix, Bozeman, USA).

### Structural variation in canola *FT* paralogs

We obtained sequence information for *FT* paralogs from a whole-genome resequencing data for the 21 canola accessions, which will be described elsewhere (Raman et al., *in preparation*). These 21 accessions also included the parental lines (Skipton and Ag-Spectrum) of the SAgS mapping population used in this study (Additional file 2: Table S2). Variation across the *FT* paralogs was extracted using the gene model information or by manually identifying gene regions based on BLAT homology (Additional file 4: Table S4). The physical positions of different *FT* paralogs (NCBI GenBank accessions; genomic sequences: FJ848913 to FJ848918; promoter sequences: JX193765, JX193766, JX193767, JX193768) were confirmed with those of the sequenced *FT* genes on the ‘Darmor’ assembly as well as with published literature [24, 25, 46]. For each accession, the *FT* nucleotide sequences were aligned using MUSCLE as implemented [47] in the software package Geneious (https://www.geneious.com) Structural variation, number of polymorphic sites within the gene and the promoter region was identified using ANNOVAR [48]. The diversity indices were calculated using the MEGA version 6 [49]. The Tajima [50] and Fay and Wu [51] tests were conducted to examine whether the frequency spectrum of polymorphic nucleotide mutations conformed to the expectations of the standard neutral model. The effect of InDel mutations on functional domains was investigated using information from the NCBI conserved domain database.

## Results

### Natural variation in flowering time across diverse environments

**Fig. 2:**
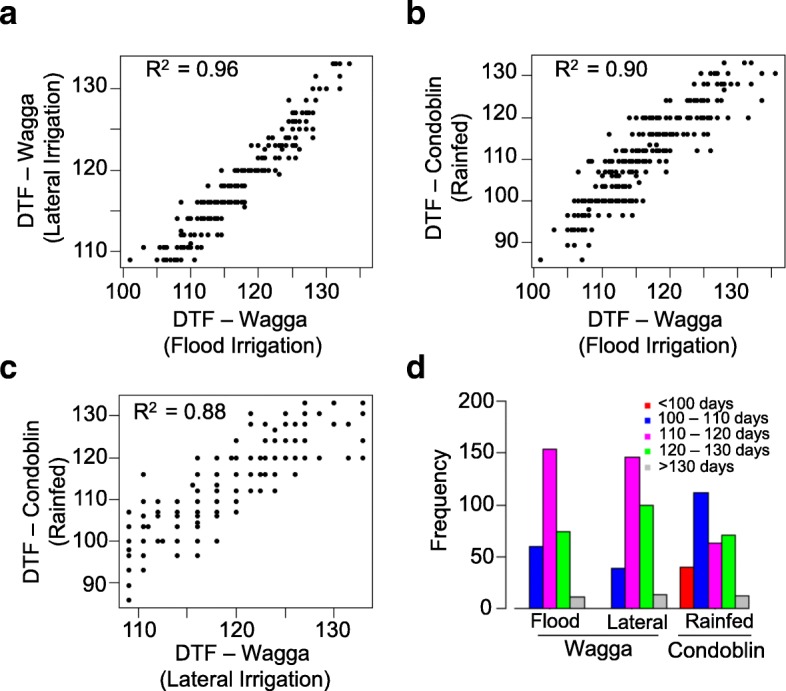
Pearson correlation for flowering time among 300 accessions of canola evaluated in field plots across different environments. Flowering time (days to flower, DTF) was assessed thrice in a week. **a** Flowering time correlation between field trials that were irrigated with lateral move or via flooding. **b** Flowering time correlation between field trials at flood irrigated plots at Wagga with rainfed plots at Condobolin. **c** Flowering time correlation between laterally irrigated plots at Wagga and rainfed plots at Condobolin and **d** Frequency distribution of canola accessions based on the days to first flower under the varied conditions

We determined the natural variation in flowering time of diverse accessions across five different environments. Across all environmental conditions, we found extensive variation in flowering time, which ranged from as little as 29.2 days up to more than 137 days (Fig. 1, Additional file 5: Table S5 and Additional file 6: Table S6). Diverse accessions grown under LD conditions (16 h light at 20 °C) typically flowered earlier (29.2 to 100.6 days) than those grown in either SD (54.3 to 131.5 days under 8 h light at 20 °C in growth cabinet) or field conditions (85.2 to 137.1 days). Accessions grown under rainfed conditions (Condobolin site) flowered earlier compared to those grown at the irrigated Wagga Wagga sites (Additional file 6: Table S6). Most of this variation was genetically controlled as the broad sense heritability (*h*^*2*^, also called as reliability) ranged from 45 to 97% across different environments (Additional file 7: Table S7). We observed positive genetic correlations (*r* = 0.88 to 0.96) for flowering time between the different field trials, suggesting that majority of the genetic variation and underlying mechanisms are shared across environments (Fig. 2).

### Flowering time variation in canola is largely due to photoperiodic response

Under controlled environmental conditions in growth cabinets, LD photoperiod substantially promoted flowering (27.6 to 77 days) (Additional file 5: Table S5, Fig. 1), while only 23.8% of accessions (*n* = 86) flowered under short days, suggesting that extended photoperiod is required for flowering. Analysis of photoperiodic response in accessions enabled us to identify specific accessions of interest, with robust photoperiod sensitive or insensitive behavior (Fig. 1, Additional file 5: Table S5). Only a small proportion (6.25%, *n* = 23) of accessions did not flower within 100 days under LD conditions. None of the winter type accessions (e.g., 03-P74, Azuma, Beluga, Ding10, Erglu, FAN28, FAN168, Gundula, Haya, HZAU-1, Maxol, Primor, Rangi, Norin-19, Tower, ZY002, ZY14, Zhongshuang-4, Zhongyou 8) flowered either in LD or in SD condition, reconfirming that vernalisation is essential for flowering in those accessions. This is consistent with these genotypes being winter/semi-winter types that typically require vernalisation to flower [12]. To assess whether there is any differential photoperiodic response, we compared the effects of photoperiod on flowering time of the accessions grown in controlled environment cabinets. Four accessions, 9X360–310 (BC15278), Georgie (BC15289), CB-Tanami (BC52411) and Hylite200TT (BC52662) had atypical flowering response, suggesting genotype x environment interactions (Additional file 1: Table S1b, Additional file 19: Figure S1).

### Relationship between flowering time and other traits

**Fig. 3:**
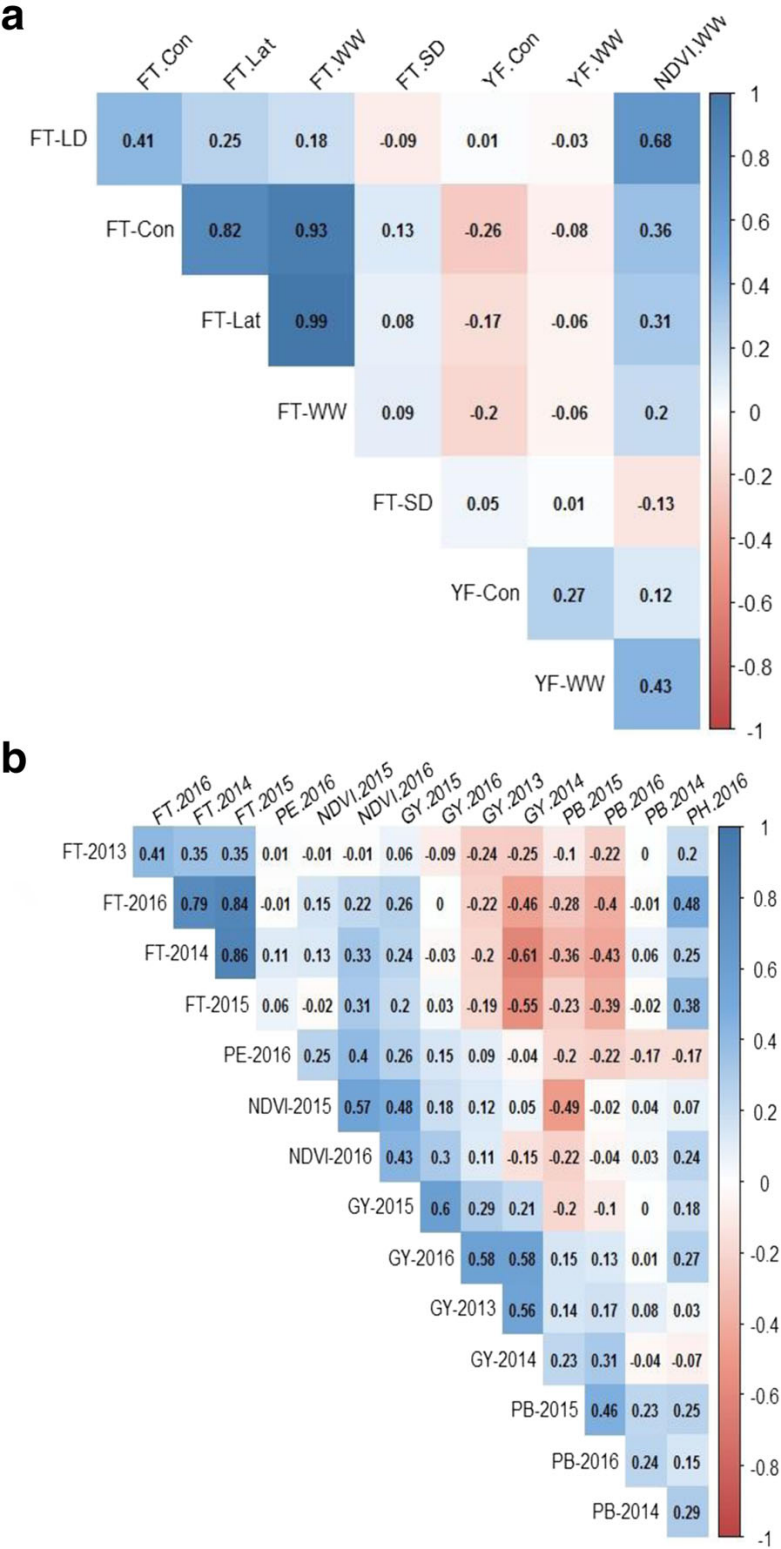
Pearson correlation between flowering time (FT) and yield related traits in a GWAS panel (**a**) and DH population derived from Skipton/Ag-Spectrum//Skipton (**b**). FT-LD: flowering time under LD conditions (days to flower); FT-SD: flowering time under SD conditions (days to flower); FT-Con: flowering time at Condobolin; FT-Lat: flowering time at Wagga (lateral move); FT-WW: flowering time at Wagga (rainfed); YF-Con: Grain yield at Condobolin; YF-WW: Grain yield at Wagga (flood irrigation), NDVI.WW: Normalised Difference Vegetative Index at Wagga; PE: plant emergence; GY: grain yield; PB: plant biomass (g/plant) and PH: plant height (cm)

To determine whether there is any relationship between flowering time and yield-related traits in canola, we analysed their genetic correlations (Fig. 3). There were low genetic correlations between the flowering time and other agronomic traits, which suggests that the growth environment play an important role in trait expression. Flowering time showed a negative correlation with grain yield across sites (WW-Wagga Wagga and Con: Condobolin) under LD photoperiodic conditions (field and controlled environments). Early vigour (NDVI.WW) showed positive correlations with flowering time (*r* = 0.2 to 0.7) under LD and field conditions (WW-Wagga and Con), and with grain yield (*r* = 0.1 to 0.4) depending upon growing environment.

### Genetic relatedness among accessions in the GWAS panel

**Fig. 4:**
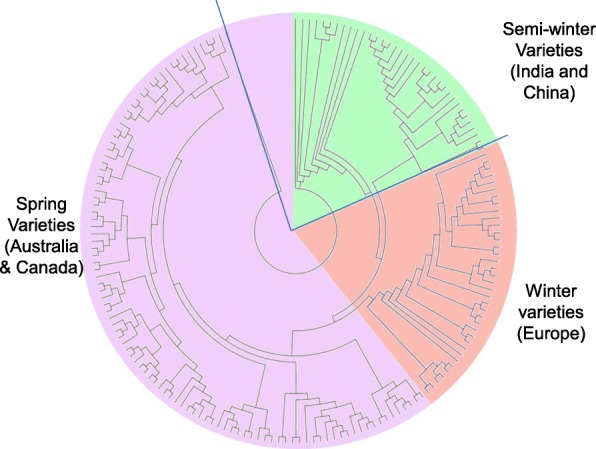
Molecular diversity in a GWAS panel of 368 *Brassica napus* accessions. Three dominant clusters shown in different shades; violet, red and light green colors represent predominantly spring, winter and semi-winter accessions of Australian, European, and Indo-Chinese origins, respectively. Details are given in Additional file 1: Table S1. Tree was drawn with MEGA 6 package [48]

SNP marker distribution across genome is shown in Additional file 20: Figure S2. SNP markers were distributed un-evenly: most were located on chromosomes A03, A07, C03, and C04 (> 780 markers/chromosome). The lowest marker density was observed in chromosome C09. A total of 11,804 SNP markers anchored to the reference *B. napus* genome, with the mean marker density of 621.3 per chromosome provided coverage of ~ 84.7 kb/marker. Cluster analysis revealed at least three main clades among accessions, representing European winter, Australian semi-spring/Canadian spring, and semi-winter of Indian/Chinese origin (Fig. 4, Additional file 21: Figure S3). The first three principal components (PC1 = 38.1%, PC2 = 11.9%, and PC3 = 5.67%) accounted for 55.7% of the genetic variation and the grouping of accessions reflected the cluster analysis (Additional file 22: Figure. S4). To estimate the extent of genome-wide linkage disequilibrium (LD) we calculated the squared allele frequency correlations (average *r*^*2*^) for all pairs of the anchored SNPs using an LD sliding window of 500 as 0.02 (Additional file 23: Figure S5). The kinship coefficient among accessions ranged from 0.03 to 0.99 suggesting a wide-range of familial relatedness between pairs of accessions (Additional file 8: Table S8), as observed in our previous study [12].

### Genetic architecture of flowering time and photoperiod response

**Fig. 5:**
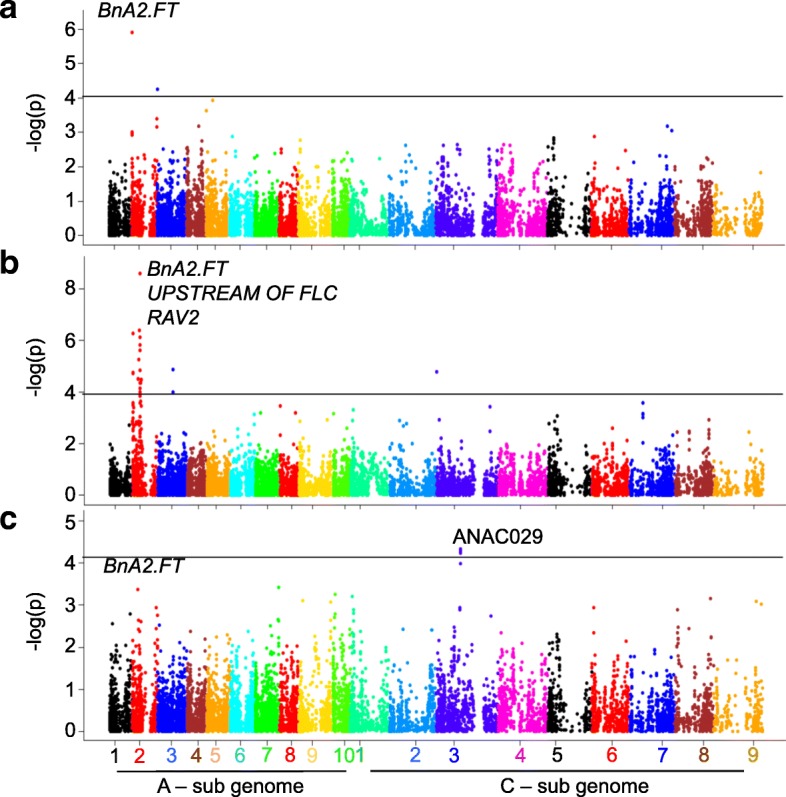
Manhattan plots for the detected associations for flowering time. Plots show genome-wide *P* values for associations between SNP markers and flowering time: **a** Field condition at Condobolin, Australia, **b** long-day conditions in controlled environnent cabinet and **c** response to photoperiod. Different colors represent different chromosomes of *B. napus* (A1-A10, C1-C9). The solid horizontal line (in black colour) signifies the threshold for significant associations - log10(*p*) value of ≤ 4

Accounting for both population structure and kinship information, we detected a total of 142 significant associations (at the genome-wide significance thresholds of LOD score of ≥3) for flowering time in diverse environments [(under field, three experiments), LD and SD conditions)]. The markers with significant associations were distributed across all chromosomes except A01 (Additional file 9: Table S9). Majority of the associated SNPs (70%) were identified on A_n_ subgenome (Additional file 10: Table S10), suggestive of an uneven distribution on the physical locations of Darmor assembly. Most of the associated SNPs (33.1%) were on chromosome A02 (47 SNPs), followed by 9.15% on C03 (13 SNPs), and these could explain the majority of allelic variation for flowering time in canola. We identified 22 unique SNP markers that accounted for associations that were detected at least in 2 different environments (Additional file 9: Table S9). Of the 142 significant associations, six SNPs crossed the Bonferroni threshold for flowering time in LD conditions, all of which are located on chromosome A02 (Table 1). Two of these SNPs (Bn-A02-p9371948 and Bn-A02-p9371633) associated with flowering time under LD conditions were located near the *FT* locus (~ 0.64 Mb, *BnA02.FT*, *BnaA02g12130D*) (Fig. 5a-c). Under different environmental conditions, we detected different associations; several of these SNP associations were mapped near the vicinity of genes known to play a regulatory role in *FT* expression in *A. thaliana* such as *FLC4, UPSTREAM OF FLC, CO, MSI1, LD, MAF4* on A02*; BnFLC3a, CO* and *EMF2* on A03; *NY-YB8* on A04; *GI* on A08; *EMF2* and *CRY2* on A10*,* and *CIB1* on C08 (Additional file 11: Table S11). We also identified 28 SNPs that showed significant association above a LOD of 3 with response to photoperiod identified under controlled environment cabinet conditions on chromosomes A01, A02, A07, A09, A10, C01, C03, C06, C08 and C09 (Additional file 11: Table S11, Fig. 5c).

**Table 1:** Genome–wide highly significant SNPs associated with variation in flowering time and photoperiodic response in diverse accessions of *B. napus*. Photoperiod response was evaluated under long (LD) and short day (SD) conditions in the controlled environment cabinet (CE). QTL marked with * were detected in the SAgS (Skipton/Ag-Spectrum/Skipton) DH population (Raman et al. 2013 [8], 2016 [12, 13, 35])

Growth Condition	Experiment site	SNP	Chromosome	Physical Position on *B. napus* cv. Darmor assembly	*P*. value for genetic association	R^2^ (%)	Physical Distance from candidate gene (Mb)	Putative Candidate gene	Other flowering time QTL found within 200 Kb regions
LD (CE)	Wagga Wagga	Bn-A02-p1232964	A02	147990	5.32E-07	4.005696	0.014152	*UPSTREAM OF FLC*	Wagga (Field)
SD (CE)	Wagga Wagga	Bn-A02-p1232964	A02	147990	1.13E-06	6.162398	0.014152	*UPSTREAM OF FLC*	Wagga (Field)
Field (plots)	Condobolin	Bn-A02-p1232964	A02	147990	1.25E-06	6.558129	0.014152	*UPSTREAM OF FLC*	Wagga (Field)
LD (CE)	Wagga Wagga	Bn-A02-p10020231	A02	6858767	4.01E-07	4.096034	0.482858	*FT (BnaA02g12130D)*	*DTF-RV (GH), Biomass 2015 (SAgS DH), Qdtf(f/s).wwai-A2a-SAgS DH
LD (CE)	Wagga Wagga	Bn-A02-p10096185	A02	6922499	1.47E-06	3.683964	0.54659	*FT (BnaA02g12130D)*	*DTF-RV (GH), Biomass 2015 (SAgS DH), Qdtf(f/s).wwai-A2a-SAgS DH
LD (CE)	Wagga Wagga	Bn-A02-p10176579	A02	7019192	2.48E-09	5.754962	0.643227	*FT (BnaA02g12130D)*	*DTF-RV (GH), Biomass 2015 (SAgS DH), Qdtf(f/s).wwai-A2a- SAgS DH
LD (CE)	Wagga Wagga	Bn-A02-p10485644	A02	7344509	7.38E-07	3.901669	0.525739	*RAV2*	LD (CE)
LD (CE)	Wagga Wagga	Bn-A02-p10493685	A02	7351405	2.34E-06	3.536863	0.519263	*RAV2*	LD (CE)
Field (single row)	Wagga Wagga	Bn-A03-p471570	A03	373818		6.217928	0.140957	*TFL1*	Field (single row), Field plots

To identify potential candidates involved in the photoperiod response, we compared the physical positions of 28 significant SNP associations for photoperiod with the physical positions of flowering time genes (Additional file 11: Table S11). Seven significantly associated SNP markers map in the vicinity (0.2 Mb) of *SPA3* (A01), *PRR5* (A02), *MAF4* (A02), *ASH1* (A07), *POWERDRESS* (A10) and *ELF6* (C09), genes underlying photoperiod response in canola accessions. The genes *ANAC029*, *EFF6*, *ABF2*, *FVE*, and *PAF1* were also identified in CE experiments and *ANAC029*, and *ASH1*, were also identified under field experimental conditions (Additional file 24: Figure S6; Additional file 11: Table S11). Consistent with our previous study (Raman et al. 2016a), our results reinforces that while the major players of flowering time appear to be conserved between Arabidopsis and canola, the specific functional roles of the paralogs might differ depending on the environmental conditions.

### QTL analysis in biparental population identifies loci for flowering time and productivity traits near *FT* paralogs

**Fig. 6:**
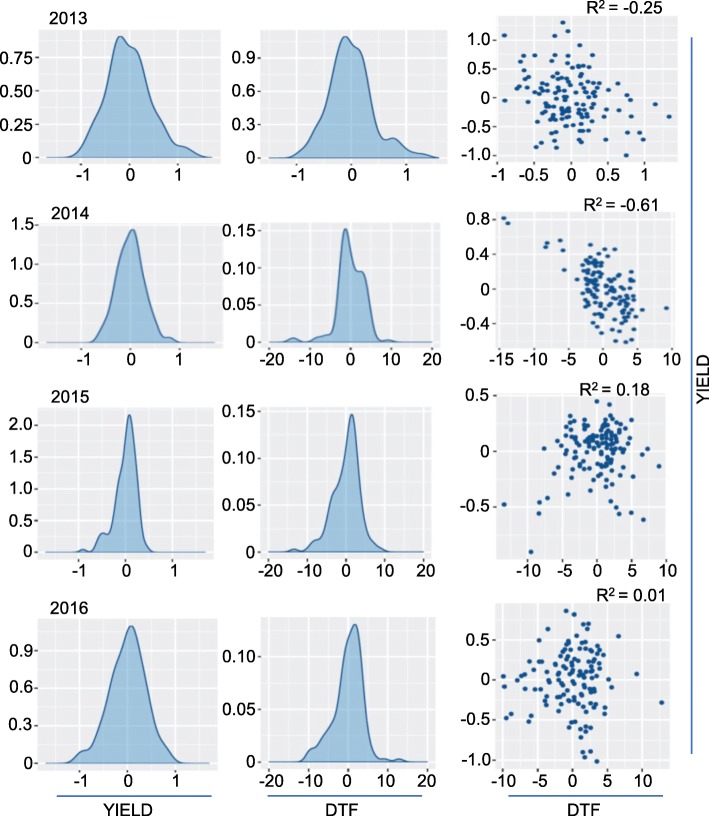
Distribution of flowering time variation in the biparental mapping population. Pair-plots showing genetic correlation of EBLUPS (empirical best linear unbiased estimators) from the univariate analysis of flowering time and grain yield among 144 doubled haploid lines of *B. napus* population derived from Skipton/Ag-Spectrum//Skipton. DH lines were grown across 4 phenotypic environments (2013–2016) in field plots, 2013 at Euberta, NSW, Australia; 2014 at Wagga Wagga, NSW, Australia [13]), 2015 and 2016 at the Wagga Wagga (this study)

To ensure capturing the relevance of entire genetic architecture of flowering time variation, we considered the SAgS DH mapping population derived from a BC_1_F_1_ cross between Australian spring type cultivars; Skipton (less responsive to vernalisation) and Ag-Spectrum (more responsive to vernalisation). We had previously utilised this cross for genetic analyses for range of traits of interest [8, 13, 35, 52–54]. The frequency distributions of the DH lines for different traits evaluated are shown (Additional file 25: Figure S7). The DH lines exhibited high broad sense heritability values (56.7 to 99%) for all traits, except for NDVI and plant emergence (29.2 to 44.3%) across environments (Additional file 12: Table S12a). There was moderate to high genetic correlations for flowering time, early vigour, plant biomass and grain yield across environments (phenotyping years) in the SAgS DH population (Fig. 6). Flowering time showed generally negative correlations with grain yield and plant biomass, whereas it showed positive correlation with early vigour and plant height. We identified several QTLs associated with flowering time, plant emergence, shoot biomass, plant height, and grain yield across phenotypic environments in the SAgS population (Additional file 12: Table S12b).

**Fig. 7:**
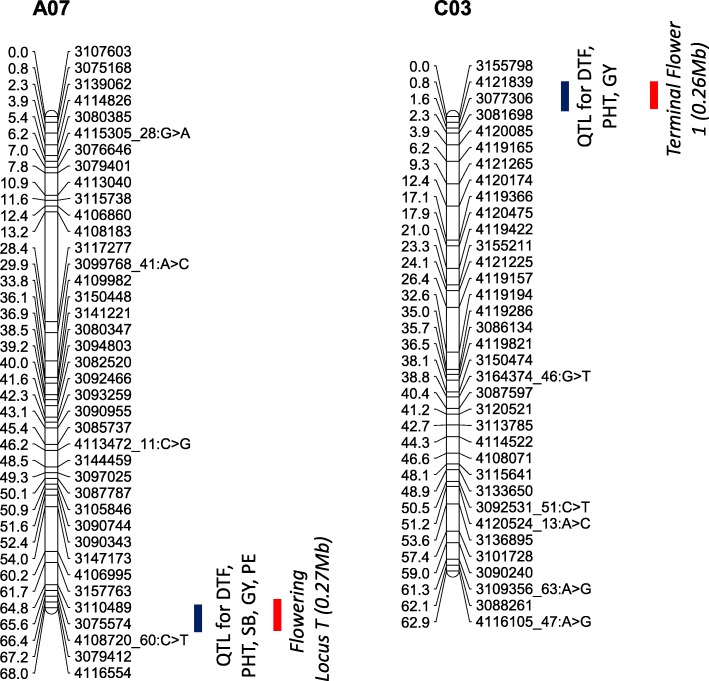
Graphical representation showing localisation of multi-trait QTL for plant emergence (PE); above ground shoot biomass (SB); flowering time (days to flower, DTF); plant height (PHT) and grain yield (GY) in a doubled haploid population from Skipton/Ag-Spectrum//Skipton. DArTseq markers and their genetic map positions are shown on right- and left-hand side, respectively. Solid lines (in blue and red colour) represent to markers that showed significant associations with traits of interest. Map distances are given in cM and displayed using the MapChart

Since we detected moderate to high genetic correlations in this population between multiple traits including flowering time (Additional file 13: Table S13), we considered whether the QTLs underlying these multiple phenotypes co-localise onto the physical map of *B. napus*. Genetic and physical localisation of markers on Darmor reference genome [44] revealed that three significant, co-located, QTLs associated with multiple traits (Fig. 7). A multi-trait QTL flanked by markers 3110489 and 3075574 for plant emergence, shoot biomass, flowering time, and grain yield mapped on chromosomes A07 was located within 0.65 Mb of the *FLOWERING LOCUS T* (*FT*, NCBI accession FJ848914.1); *BnA2.FT* paralog in *B. napus* [24]. Consistent with GWAS analysis, we detected QTLs near the *FT* in the biparental population (Fig. 7). Mapping of pleiotropic trait QTL in the vicinity of *FT* (A07) suggest that *FT* may have multifaceted role in plant development and productivity traits.

### Expression levels of *FT* paralogs explain significant variation in flowering time

**Fig. 8:**
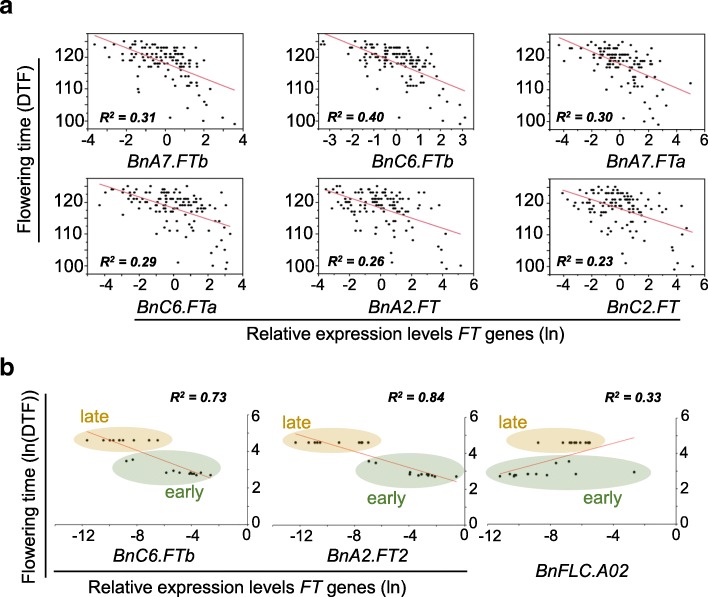
*FT* is a major determinant of flowering time variation and photoperiod gene in canola varieties. **a** Expression analysis of different *FT paralogs (BnA2.FTa, BnC2.FT, BnA7.FTa, BnA7.FTb, BnC6.FTa, BnC6.FTb)* on leaves taken from field grown plants of 144 doubled haploid lines of Skipton/Ag-Spectrum//Skipton, and its correlation with flowering time. **b** Expression analyses of *FT* genes; *BnC6.FTb (*chromosome C6) and *BnA2.FT* (chromosome A02) and *BnFLC2* on leaves taken from LD grown plants of 24 diverse accessions, representing flowering time diversity in a GWAS panel. The relative expression levels of *FT* and *FLC* after normalisation with the reference *UBC9*, is plotted against flowering time

To assess whether changes in the expression of different *FT* paralogs could explain the phenotypic variation in flowering time, we examined expression of *FT* paralogs among field-grown plants of all 144 DH lines. Expression levels of all 6 *FT* paralogs displayed significant association with flowering time (*p* < 0.001), with different copies accounting for genetic variation in flowering time variably; ranging from 23% (*BnC2.FT*) to 40% (*BnC6.FTb*) (Fig. 8a). The *FT* homologues *BnA7.FTb* and *Bn*A7*.FTa* localised near a multiple trait QTL (Additional file 12: Table S12) could explain 30 and 31% of genetic variation in flowering time, respectively. Sequence analyses of the PCR products also confirmed that *BnC6.FTb* and *BnA7.FTb* are accurately detected in our assays.

To further assess whether a similar pattern is also observed among natural variants, we assessed the expression of *BnC6.FTb, BnA2.FT2* and *BnFLC.A02.* We choose *BnC6.FTb* because it showed the highest correlation in the DH population. *BnA2.FT2* was detected as a QTL in the diversity set of 24 accessions, whilst *BnFLC.A02* was identified in accessions that differed significantly in their flowering time. Consistent with the QTL analysis and the expression studies in DH populations, we observed significant differences in *FT* and *FLC* expression that correlated with flowering time among 24 diverse accessions selected on the basis of flowering time diversity (Fig. 8b). Consistent with the timing of sample collection (i.e., just prior to flowering), we detected expression variation in *FT* rather than *FLC* accounting for most of the flowering time variation in these diverse set of 24 accessions. Taken together this study revealed that irrespective of the causal variation, the phenotypic variation is associated with changes in the expression levels of the floral integrator *FT.*

To unravel the *cis* and *trans* acting candidates associated with differential *FT* transcripts expression, we first sought SNPs that affect expression levels of all *FT* homologues in diverse canola accessions. Then, we layered this information on the physical map positions of SNPs associated with genetic variation in flowering time and photoperiod response (Additional file 14: Table S14). We identified a total of 13 SNPs mapped on chromosome A07 and C03, in the vicinity of multiple trait QTLs that we identified in the SAgS population. The candidate genes located near significant SNP associations are *FT, ELF4-L2, PRR9, VIN3, BnFLC.C3b (FLC5,* AY036892.1*)*, *FPA, SPA1* and *TOE1* (Additional file 11: Table S11).

### *FT* paralogs exhibit structural sequence variation in *B. napus* accessions

**Fig. 9:**
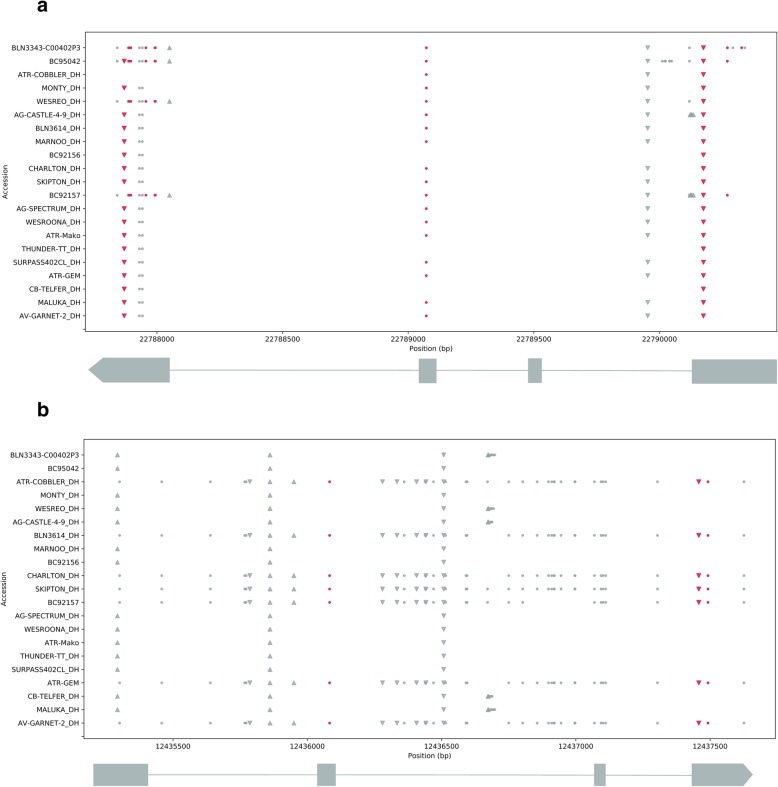
Graphical representation showing structural variation in (**a**) *Bn.A07.FTb* (*BnaA07g33120D*) and (**b**) *BnC04.FT* gene (*BnaC04g14850D*) among 21 accessions of canola. Dots represent SNPs, triangles insertions, and inverted triangles deletions. SNPs and indels shaded in red are non-synonymous. The four exon gene model is shown below each plot with the exons as grey boxes and the introns as lines. Details of sequenced accessions are given in Additional file 1: Table S1. *FT* variant used for revealing diversity in *BnaC04g14850D* among 21 accessions are given in Additional file 18: Table S18

**Fig. 10:**
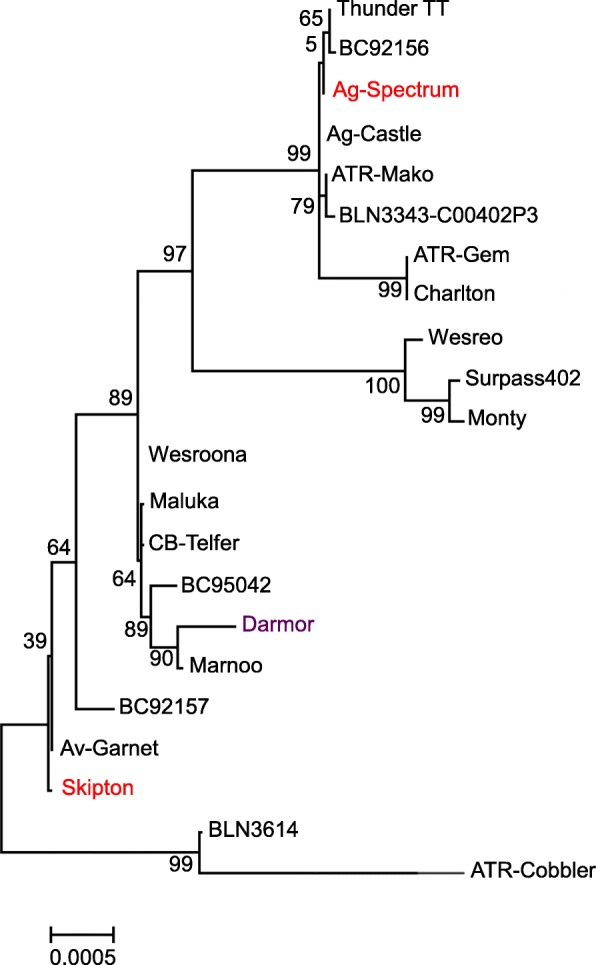
Neighbour-joining tree based on nucleotide variation across all *FT* paralogs among 21 accessions of *B. napus* representing GWAS and parental lines (shown in red color) of a doubled haploid population derived from Skipton/Ag-Spectrum//Skipton. Tree was generated in MEGA 6. Nucleotide variation in *FT* genes was also compared with the corresponding *FT* genes in the reference Darmor-*bzh*, in colour. Number refers to percent bootstrap support for branches with greater than 50% support

In total, nine *FT* copies were identified in *B. napus* accessions (Additional file 15: Table S15), including three putative *FT* copies on chromosomes A01, C02, and C04, (Additional file 15: Table S15). Sequence analyses showed considerable variation in level of synonymous and non-synonymous SNP variations, Insertion-deletions (InDel) in promoters, as well as exonic and intronic regions. A total of 310 segregating sites were detected across *FT* paralogs. Our results showed that frequency spectrum of structural variants for *BnA02.FT*, *BnC02.FT* and *BnC06.FT* conformed to neutral expectations, while *BnC04.FT* and *BnA07.FT* showed non-conformance to neutrality, suggesting evidence of selection (Additional file 16: Table S16). We detected high level of diversity in *FT* paralogs mapped on A07, C04 and C06 chromosomes (Additional file 17: Table S17, Additional file 18: Table S18). For example, *BnC04.FT* (*BnaC04g14850D*) contained 35 SNPs, with the majority (21 SNPs) located in intron II (Fig. 9). Interestingly, an 8-bp deletion of the sequence ‘TTCCGGAA’ (coordinates BnC04:12,437,458-12,437,465 bp) was observed in exon-IV of *BnC04.FT* in seven accessions; Av-Garnet, BC92157, Skipton, Charlton, BLN3614, ATR-Cobbler, ATR-Gem and in Darmor-*bzh* (reference genotype). This mutation creates a frameshift that removes the highly conserved C-terminal domain containing the PEBP-domain and several substrate-binding sites. Cluster analysis showed that all variants formed a distinct cluster (Fig. 10). In the *BnA07.FTb* (*BnaA07g33120D*) we identified two indel mutations in the coding region (Fig. 9). The first is a single nucleotide deletion in exon 4 that is heterozygous with the wild type allele in Australian varieties; Av-Garnet, Skipton, Charlton, BC92156, Marnoo, BLN3614, Ag-Castle, Monty, Maluka, BLN3343-C00402, CB-Telfer, ATR-Gem, Surpass402, ThunderTT, ATR-Mako, Wesroona and Ag-Spectrum (the remaining lines are homozygous wild-type). The deletion results in a frameshift that affects the final 20 amino acids of the encoded peptide, including the 9 amino acids of the PEBP domain. The second InDel is a 3 base-pair mutation in exon 1 (His60-deletion) that is found in all our sequenced lines. These polymorphisms are consisted with the observed QTLs in the vicinity of *FT.*

### Structural variation in *FT* promoter region

We further searched CArG box and other motifs for *FLC, SOC1, SMZ* and *CO* which can potentially bind to repress *FT* expressions [55] in introns (especially intron 1) exons and promoter regions. A putative *CO* binding site within Block A: type II = ‘ATTGTGGTGATGAGT’ (Wang et al. 2009 [24]) was found in both *BnA02.FT* and *BnC02.FT* genes. However, this Type-II block ‘A’ sequence was absent in all *FT* paralogs located on the A07 and C06 chromosomes. ‘CArG’ box (CC(A/T)6GG) was absent in introns 1 of *BnA02.FT* and *BnC02.FT* genes. We also found several ‘CACTA’ elements in *B. napus FT* paralogs. For example, in *BnaC04g14850*, a total of four motifs were identified; three were present in introns (2 in Intron 2, antisense direction, and one in sense strand), and one CACTA motif was identified in Exon-IV. In *BnA02.FT*, a total of 834 CACTA motifs were identified in promoter, intron 1 and exon II. We also identified homologous sequences to *FT* promoter blocks C and E of *A. thaliana* [56] in three *B. napus FT* genes (BnaC06g27090D, BnaA07g25310D, and BnaA02g12130D). *Block E* was also identified in BnaC06g27090D with blastn (Additional file 26: Figure S8). In comparison to the *Block C* alignments, the binding regions were not well conserved in *Block E.* The structural variants for the four *FT* genes were plotted. Finally, in order to determine whether polymorphism in *FT* directly relates to flowering time variation, we performed phylogenetic analysis of 21 accessions representing GWAS panel and parents of mapping populations being used in the Australian Brassica Germplasm Improvement Program. Our results showed that grouping for both spring and winter types based on *FT* paralogs was not that distinct (Fig. 10) suggesting that other key flowering genes such as *FLC* and *FRI* may have contributed to diversification of these morphotypes [14, 57].

## Discussion

In this study we explored the genetic architecture underlying phenotypic diversity in flowering time, an important trait involved in plant development, adaptation and productivity. Our results demonstrate that there is extensive genetically controlled natural variation in flowering time of canola. Variation in the response to photoperiod (as revealed from LD and SD conditions) appears to be another key determinant of flowering time differences among canola accessions (Fig. 1). Despite extended photoperiod at 20 °C, several accessions did not flower under CE conditions. These accessions flowered when exposed to extended periods of cold temperatures suggesting that these accessions require vernalisation [12, 13, 52]. Thus, a combination of variation in photoperiod and vernalisation response causes phenotypic diversification of flowering time in canola (Fig. 1).

In order to have a minimum effect of vernalisation on flowering time, all field trials were conducted in the middle of June (instead of April the main canola growing season in Australia). We identified a highly significant QTL close to *FT* locus on chromosome A02 for flowering time variation in field-grown or CE cabinet-grown plants, suggesting that *FT* is a major candidate for flowering time across different growing environments (Fig. 4). This QTL was also mapped within 80 kb of a QTL for vernalisation response in our previous study [12], suggesting that *FT* integrates signals from both photoperiod and vernalisation pathways and regulates the transition from vegetative to reproductive phase in canola.

The functional role of *FT* was determined using quantitative RT-PCR using six *FT* paralog specific primers. Our results demonstrated that all paralogs underlie genetic variation in flowering time in canola. For the first time, we show *FT* expression in a canola population grown under field conditions is significantly associated with variation in flowering time. It was interesting to observe that most of variation in flowering time was explained by A02 locus in a GWAS panel, and A02 and A07 loci near *FT* paralogs in the SAgS DH mapping population (Fig. 6, Additional file 12: Table S12). However, the maximum correlation (*R*^*2*^ = 0.4) was observed for *BnC6.FTb* homologue, followed by *BnA7.FTb* (*R*^*2*^ = 0.31), *BnA7.FTa* (*R*^*2*^ = 0.30), *BnC6.FTa* (*R*^*2*^ = 0.29), *BnA2.FT* (*R*^*2*^ = 0.26), and *BnC2.FT* (*R*^*2*^ = 0.23). Higher correlation among different paralogs suggested that different copies can substitute allelic effect on flowering time. Unlike previous studies [25, 26], our results suggest that all copies of *FT* may be functional. Although all *FT* paralogs except *BnC6.FTa* and *BnC6.FTb* map at the same physical position as the closest relative of *FT*, *TWIN SISTER OF FT* (*TSF*), cloning of six paralogs of *FT* in canola [24, 25] discounted the possibility of *TSF* controlling variation in flowering time which is shown to have much lower expression levels than *FT* [58–60].

We detect considerable structural variation in promoter, as well as in exonic and intronic regions in *FT* genes located on chromosomes other than A01 and C02. These high levels of polymorphism suggest that the *FT* gene is a major target for selection during domestication and systematic breeding of canola. *FT* is a member of the PEBP family and multiple paralogs have evolved from its common ancestral species, however these paralogs may have retained, lost or gained gene function in the polyploid genome of canola [24, 61]. Our sequencing analyses reveals that different copies of *FT* harbour mutations including in the *CArG*, *CACTA, Block C* and *Block E* - the binding sites for the transcriptional factors such as FLC, SVP, GI, CO, CIB, CRY2 and SMZ proteins (Additional file 24: Figure S6), which regulate of the expression of *FT* [1, 25, 56, 62, 63]. Mutations in *FT* and *TFL1* paralogs in canola have been reported to affect flowering and yield components [26]. Mutants or isogenic lines carrying different *FT* paralogs and/or their combination are required to establish the precise role of each paralog in both vegetative and reproductive phase of plant development. While our expression analyses of *FT* genes hints at functionality of these paralogs, further research is required to establish whether there is any role of transcriptional enhancers: *Block C* and *Block E* on the *FT* expression [56] as well as its association with other traits of agronomic interest.

We show that *FT* has multifaceted role in diverse traits that influence plant development. QTLs for several traits such as plant emergence, early vigour, plant biomass, plant height, grain yield, were localized with flowering QTL in a cluster and the expression level of *FT* showed a good association with different traits. However, this relationship was dependent upon G × E interaction (Additional file 19: Figure S1). These findings hint that flowering time, driven by *FT* paralogs have variable influence on different traits under different environments. However, it was difficult to establish in this study due to presence of multiple copies of *FT* in canola genome.

This study demonstrates multigenic inheritance of flowering in the SAgS population. However, a relatively small size population (*n* = 144) may have compromised the estimates of QTL identified herein. In addition, QTL only accounted for small genetic effects (2.7 to 10.3%) in this study (Additional file 12: Table S12). This is in contrast with other studies, which reported major QTLs for flowering time [64]. Recently, Tyagi et al. [65] showed that Brassica *FT* homeologs influence flowering time, branching pattern, plant height, silique length and width, seed size, stomatal density, and fatty-acid profile in *B. juncea*. Our expression analyses revealed that enhanced *FT* gene expression is related with early flowering in the doubled haploid lines of Skipton/Ag-Spectrum//Skipton (Fig. 8). In a previous study, Raman et al. [13] showed that early flowering DH lines having Skipton QTL alleles yield higher than those having late flowering allele (Ag-Spectrum). These results suggest that canola varieties having higher *FT* gene expressions can be selected for enhancing productivity.

In canola, sequence variation in *BnFLC.A10* appears to underlie QTL for both flowering time as well as root biomass [21, 66]. In addition, flowering time has been implicated in plasticity of water-use efficiency, carbohydrate availability, plant vigour, resistance to diseases and yield [67–70]. We propose that alleles that showed significant association with flowering time and grain yield in the water-limited years experienced in 2013 and 2014, are of high relevance even though they did not reveal genetic associations in water-unlimited years (non-stress environment, 2015 and 2016). Environmental stress tends to drive changes in flowering time in Brassica as a result of change in allele frequencies of the flowering time genes [71, 72]. Our data also suggest that different *FT* paralogs regulate flowering time depending upon environment. For example, QTLs for flowering time were identified close to BnaA07g25310D in 2013 and 2014, however a QTL for flowering time was mapped on chromosome C04, close to a different *FT* paralog, *BnaC04g14850D* in 2015 and 2016. Since, flowering time showed a good correlation with plant emergence, early vigour, shoot biomass, and grain yield; and enhanced *FT* expression is also correlated with early flowering, it is possible that *FT* may be one of the drivers promoting early growth in canola, therefore contributing to higher grain yield in canola especially under terminal drought and heat stress environments prevalent in Mediterranean countries.

The findings presented here reveal that the genetic architecture of natural variation in flowering time involves multiple alleles having major effects located near *FT*, *UPSTREAM* of *FLC* and *RAV2* paralogs on chromosome A02 (Table 1, Additional file 24: Figure S6). This is in contrast to genetic variation in flowering time regulated by vernalisation which is controlled by multiple alleles distributed across genome [8, 10, 12]. Both positive and negative regulators of *FT* were located near significant SNP associations; for example, under LD treatment *FLC* that repress the *FT* transcription by direct binding to the CArG sites in intron 1 and promoter region of *FT* was detected [55]. The role of the candidate genes: *GI*, *FD*, *SAM*, *AGL18*/*FUL* in flowering time is well documented [7]. We also identified significant SNP associations for flowering time in the vicinity of *H*^+^*-ATPse* (Additional file 24: Figure S6) which is implicated in stomatal opening and enhanced *FT* expression in the guard cells [28]. In addition, *MSI*, *EMF2*, *FVE*, and *CURLY LEAF* which regulate *FT* transcription via trimethylation of H3K27me3, H3K4me3 and EARLY FLOWERING 6 [1, 56] were located in the vicinity of significant SNPs. These results suggested that both approaches utilized in this study: QTL as well as GWAS analyses are suitable for revealing the genetic architecture of flowering time in canola.

Based on their photoperiodic response, all genotypes could be grouped into photoperiod sensitive, photoperiod insensitive (less sensitive), and non-flowering types (vernalisation sensitive). Classification of such genotypes based on flowering habit was also supported with our molecular marker clustering, which placed the majority of the winter type varieties from Europe, China and Japan, in a single cluster (cluster II, Additional file 21: Fig. S3). These results supported that spring (semi-spring in Australia), semi-winter and winter canola belong to distinct genepools. A number of semi-winter accessions from China grouped into separate clade. Previous research has shown that Chinese canola germplasm is derived as a result of intensive crossing between winter canola introduced from Europe via Japan and spring type *B. rapa* for local adaptation [73].

In summary, we have demonstrated through a series of complementary and exploratory analyses based on association tests using genome-wide SNPs, expression QTL and quantitative RT-PCR that the natural variation in flowering time and response to photoperiod revealed in this study is controlled by *FT* and other loci dispersed across the genome, and modulated by the environment. GWAS approach delineated genomic regions and provided insights into the genetic architecture of flowering time and its multifaceted role in plant development and productivity traits. Although some alleles identified in this study may not be causative of phenotypic differences in flowering time, they still represent valuable selection tools to increase rate of genetic gain in canola improvement programs. Several Illumina Infinium™ SNP and *FT* gene specific markers located near the QTL associated with trait variation and known flowering time genes [74–76] would enable the identification of canola accessions with optimal *FT* expression and agronomic trait performance. Further research is required to understand the role of different *FT* copies in canola productivity across target environments.

## Additional files

Additional file 1: **Table S1.** Accessions used to assess natural variation in flowering time and photoperiodic response. (XLSX 24 kb)

Additional file 2: **Table S2.** Details for phenotyping, experimental designs and QTL analysis (DOCX 314 kb)

Additional file 3: **Table S3.** Mean marker density of Illumina SNP markers genotyped in a canola GWAS panel of 368 accessions. (XLSX 8 kb)

Additional file 4: **Table S4.** PCR primers used for expression analysis by RT-qPCR (Guo et al. 2014) (XLSX 12 kb)

Additional file 5: **Table S5.** *Brassica napus* genome BLAT HITs against the *Arabidopsis thaliana FLOWERING LOCUS T* (AT1G65480.1, RSB8/FT/chr1:24331428–24333935) using Darmor reference assembly (http://www.genoscope.cns.fr/blat-server/cgi-bin/colza/webBlat). *FT* paralogs identified in a previous study (Schiessl et al. 2014 [45]) are also shown for comparison. (XLSX 28 kb)

Additional file 6: **Table S6.** (A) Natural variation in flowering time in a GWAS panel of 368 lines of *B. napus* grown under controlled environment cabinets under short day (8 h light and 16 h dark) and long day (16 h light and 8 h dark); (B) Natural variation in flowering time in a GWAS panel of 300 lines of *B. napus* grown under field conditions. - represents to missing data and (C) Broad sense heritability of flowering time under controlled and field condition among canola accessions. (XLSX 8 kb)

Additional file 7: **Table S7.** Marker LD across *B. napus* genome. (CSV 1575 kb)

Additional file 8: **Table S8.** Familial relationships between pairs of accessions used for GWAS. (XLSX 41 kb)

Additional file 9: **Table S9.** Marker trait association identified for flowering time and photoperiodic response in a GWAS panel of canola. Response to photoperiod was assessed under controlled environment conditions, LD: Long day conditions (16 h light, 8 h dark at 20 degree); SD (8 h light, 16 h dark at 20 degree). Flowering time was also evaluated under field conditions at three sites: Wagga Wagga (irrigation, NSW, Australia), Wagga Wagga (lateral move irrigation site) and Condobolin (rainfed site, NSW, Australia) Days to flowering was used for GWAS analysis using GAPIT program in R and Golden Helix (SVS, with and without principal component analysis). (XLSX 9 kb)

Additional file 10: **Table S10.** Distribution of significant marker associations for flowering time and photoperiod response, evaluated under controlled environment cabinets and field conditions (three sites) in a GWAS panel of canola (XLSX 936 kb)

Additional file 11: **Table S11.** Candidate gene associated with flowering time and photoperiodic response in the GWAS and DH population. (XLS 35 kb)

Additional file 12: **Table S12.** Significant QTL associated with flowering time and grain yield identified in a doubled haploid population derived from a single BC_1_F_1_ from the Skipton/Ag-Spectrum//Skipton population grown in four environments, at Euberta (2013) and Wagga Wagga (2014, 2015 and 2016). QTL in bold are repeatedly detected across environments/traits. QTL in bold and italics are multi-trait QTL (pleiotropic). (DOCX 23 kb)

Additional file 13: **Table S13.** Genetic correlation between different traits measured in the doubled haploid population from Skipton/Ag-Spectrum//Skipton across environments. (XLSX 15 kb)

Additional file 14: **Table S14.** Genome-wide association analysis (eQTL) showing statistical association between Illumina SNP markers and expression data of *BnC6.FT* gene in 300 accessions of *B. napus*. Linear marker regression analysis was performed in the SVS package (Golden Helix). (XLSX 11 kb)

Additional file 15: **Table S15.** Gene structures of different *FT* paralogs identified in the resequence data from 21 accessions of *B. napus* (test samples). Exon/intron genomic coordinates of the *B. napus* reference cultivar are based on the current gene models (annotation version 5). Numbers in the table represent lengths in base-pairs. Exon/intron length variation in the 21 accessions (in bold) is only counted for InDels that are homozygous. (XLSX 13 kb)

Additional file 16: **Table S16.** Summary of structural and polymorphic variation identified among 21 *B. napus* accessions representing GWAS and validation population used in this study. Numbers in table represent counts of unique variants observed across the 21 accessions. Abbreviations: SNV: structural nucleotide variant, InDel: Insertion-deletion, S = Number of segregating sites, ps = S/n, Θ = ps/a1, π = nucleotide diversity, and D is the Tajima test statistic (Tajima, 1989). (XLSX 12 kb)

Additional file 17: **Table S17.** *FT* variant used for revealing diversity in *BnaA07g33120D* among 21 accessions resequenced. (XLSX 10 kb)

Additional file 18: **Table S18.** *FT* variant used for revealing diversity in *BnaC04g14850D* among 21 accessions resequenced. (XLSX 13 kb)

Additional file 19: **Figure S1.** Canola genotypes showing G X E interactions when grown under LD and SD conditions in controlled environment cabinet. Mean flowering time is estimated in days. Details of varieties shown here represented to BC accessions (Additional file 1: Table S1). (PPTX 213 kb)

Additional file 20: **Figure S2.** Genome-wide distribution (A) and density (B) of single nucleotide polymorphisms, in a genome wide association diversity panel of 368 *Brassica napus* accessions. Regions that are rich and poor SNP density are shown in dark and whitehorizontal bars, respectively. The number of SNP markers anchoring on different chromosomes (A1-A10 and C1-C9) of the physical map of the *B.napus* genome is given on the x-axis. (PPTX 959 kb)

Additional file 21: **Figure S3.** Genetic diversity and population structure in a GWAS panel of 368 *Brassica napus* accessions. Three clusters designated as I, II and III represent predominantly Chinese, European, and Australian accessions, respectively. Details of accessions are given in Additional file 1: Table S1. (PPTX 1670 kb)

Additional file 22: **Figure S4.** Principal components (PC1 and PC2) analysis showing population structure in a GWAS diversity panel of 368 *B. napus* accessions. Three major clusters designated as I, II, and III, consistent with the cluster analysis (Additional file 20: Figure S2). (PPTX 2450 kb)

Additional file 23: **Figure S5.** The average linkage disequilibrium (LD) decays (r^2^) approach 0.02 when distance between SNPs was approximately 200 Kb. Distance in bp is shown on X-axis. (PPTX 135 kb)

Additional file 24: **Figure S6.** Candidate genes located within 200 kb from the significant SNPs associated with flowering time in a GWAS panel of canola. Accessions were grown under long day (LD, 14 h light), short day (SD, 8 h light) treatments in controlled environments (CE) and three field conditions at Wagga Wagga [in single rows: WAG-FT (Row) and plots: WAG-FT (Plots)] and Condobolin [in plots: CON-FT (Plots). Response to photoperiod was estimated as the difference between LD and SD treatments (days). Details are given in Additional file 11: Table S11. (PPTX 189 kb)

Additional file 25: **Figure S7.** (A). Frequency distribution of shoot biomass in a SAgS DH population phenotyped across 2014–2016 growing environments. (B). Frequency distribution of fractional ground cover, measured as NSVI with a hand-held GreenSeeker machine, in a SAgS DH population phenotyped across 2015–2016 growing environments). (C). Frequency distribution of days to flower in a SAgS DH population phenotyped across four environments (2013–2016). Phenotypic data of 2013 and 2014 was published previously (Raman et al. 2016 [12, 13, 52]). (D). Frequency distribution of plant height and plant emergence in a SAgS DH population phenotyped in 2016 growing environments. (E). Frequency distribution of grain yield in a SAgS DH population phenotyped across four environments (2013–2016). Phenotypic data of 2013 and 2014 experiments was published previously (Raman et al. 2016 [12, 13, 35]). (PPTX 5970 kb)

Additional file 26: **Figure S8.** A: Regions of homology between the B. napus FT regions and block C from A. thaliana. Putative binding sites are indicated based on ref . BN_chrC06 is upstream from BnaC06g27090D, BN_chrA07 is upstream from BnaA07g25310D, and BN_chrA02 is upstream from BnaA02g12130D. A corresponding block C region for BnaC02g45250D could not be identified. B: Regions of homology between the *B. napus* FT regions and block E from *A. thaliana*. Putative binding sites are indicated based on ref. . BN_chrA07 is downstream from BnaA07g25310D, BN_chrC02rnd is downstream from BnaC02g45250D, BN_chrA02 is downstream from BnaA02g12130D and BN_chrC06 is downstream from BnaC06g27090D. C: Summary of SNP and Indel variation in the B. napus FT gene BnaA02g12130D across 21 lines. The gene model is shown below the plot. Key: Insertions = triangle, deletions = inverted triangle, SNPs = dots, red = nonsynonymous change. D: Summary of SNP and Indel variation in the B. napus FT gene BnaA07g25310D across 21 lines. The gene model is shown below the plot. Key: Insertions = triangle, deletions = inverted triangle, SNPs = dots, red = nonsynonymous change. E: Summary of SNP and Indel variation in the *B. napus* FT gene BnaC02g45250D across 21 lines. The gene model is shown below the plot. Key: Insertions = triangle, deletions = inverted triangle, SNPs = dots, red = nonsynonymous change. F. Summary of SNP and Indel variation in the *B. napus* FT gene BnaC06g27090D across 21 lines (only a subset of lines are shown). The gene model is shown below the plot. Key: Insertions = triangle, deletions = inverted triangle, SNPs = dots, red = nonsynonymous change. (PPTX 3860 kb)

## Data Availability

All experimental materials are available on request (email: harsh.raman@dpi.nsw.gov.au). The sequencing data of *FT* paralogs is given in Additional file 18: Table S18.

## Abbreviations

BLAST: Basic local alignment search tool
CE: Controlled environment cabinet
DH: Doubled haploid
eQTL: Expression QTL
FT: Flowering locus T
G X E: Genotype x environment interaction
GWAS: Genome wide association study
MAF: Minor allele frequency
PCR: Polymerase chain reaction
QTL: Quantitative trait loci
SNP: Single nucleotide polymorphism
