# Supplementary material for: GWAS hints at pleiotropic roles for FLOWERING LOCUS T in flowering time and yield-related traits in canola
Source: BMC Genomics. 2019 Aug 6;20:636. doi: 10.1186/s12864-019-5964-y (PMC6685183; doi:10.1186/s12864-019-5964-y)
Supplement: Supplementary file 2 — Table S2. Details for phenotyping, experimental designs and QTL analysis (DOCX 314 kb) [file 12864_2019_5964_MOESM2_ESM.docx]

**Supplemental Table S2.**

**Layout of Wagga Wagga Field Plot Trial-2017**

**Layout of Condobolin Field Plot Trial-2017**

**Layout of Wagga Wagga Single Row Trial-2017**

**Experiment design for investigating the role of *FT* and *FLC* in flowering time under controlled cabinet**


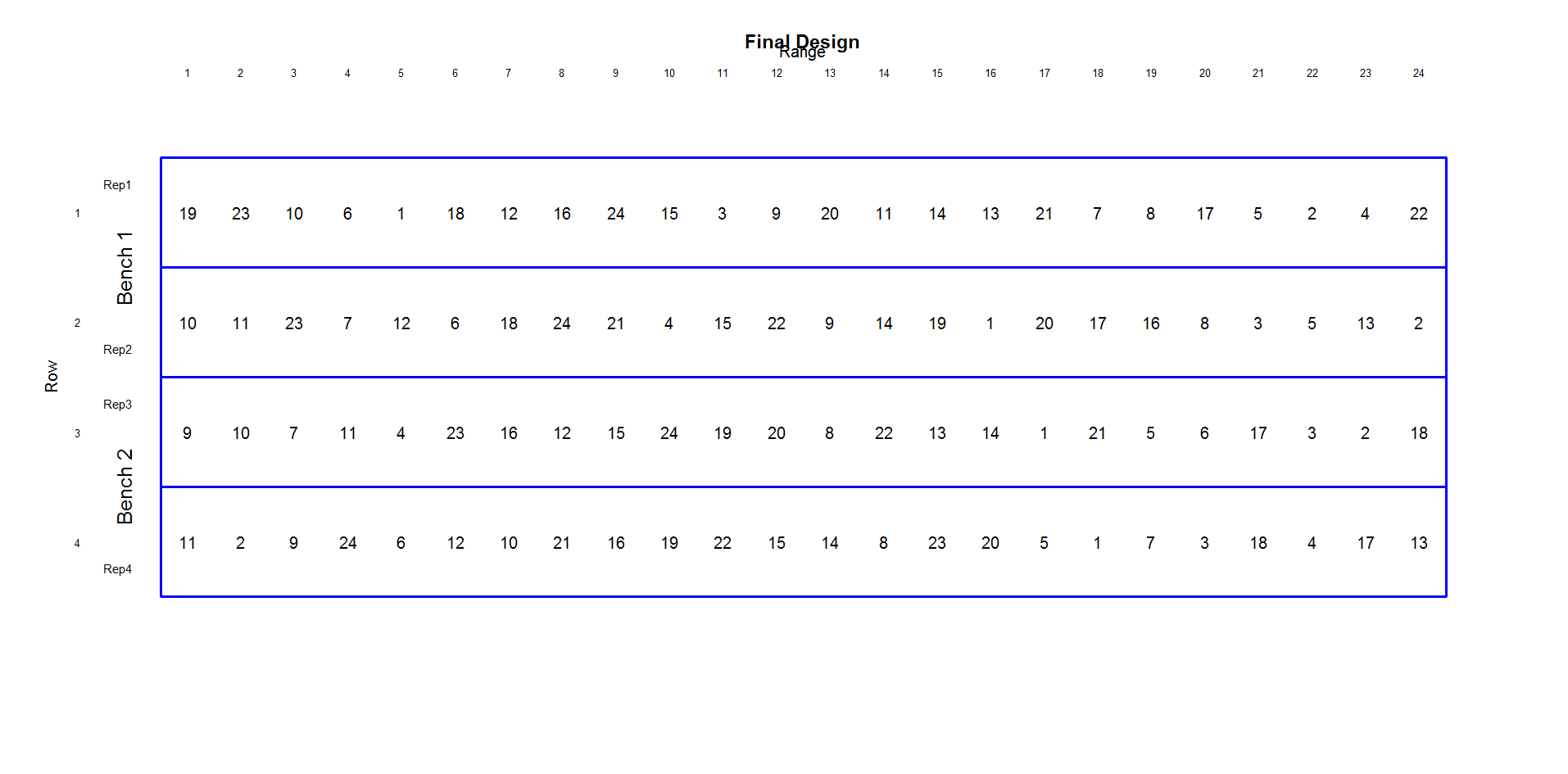


**Details of 24 diverse accessions selected from the GWAS panel used for FT and *FLC* expression analyses**

Accession name Accession Number

| ZHONGYOU-ZA-NO8_DH | 15352 |
| --- | --- |
| Beluga | 51490 |
| GSL1-1_DH | 52505 |
| ZY014-8_DH | 52570 |
| RANGI-501-12_DH | 52586 |
| PRIMOR-1_DH | 52611 |
| NORIN-19-469973-11_DH | 52612 |
| AZUMA-13_DH | 52633 |
| TOWER | 95048 |
| ERGLU | 95050 |
| WESBELL_DH | 52387 |
| TATYOON_DH | 52390 |
| CB-TRILOGY_DH | 52412 |
| ROTTNEST-TTC_DH | 52422 |
| SARDI524TT-11_DH | 52555 |
| AGT346-11_DH | 52659 |
| ATR-SNAPPER_DH | 94504 |
| CB-JARDEE-HT-16_DH | 94551 |
| Rapid cycling | 95194 |
| Columbus | 50557 |
| Darmor | 51831 |
| CB-Telfer | 52430 |
| Skipton | 52649 |
| Ag-Spectrum | 52374 |
|  |  |

**Parental lines representing the GWAS panel and genetic mapping populations of *B. napus.* These lines are being used in Australian National Brassica Germplasm Improvement Program at Wagga Wagga. The whole-genome resequencing was performed using Illumina Hiseq as outlined under materials and methods section.**

|  | Parental lines of doubled haploid mapping populations |
| --- | --- |
| 1 | AV-GARNET |
| 2 | MALUKA |
| 3 | CB-TELFER |
| 4 | ATR-GEM |
| 5 | SURPASS402CL |
| 6 | THUNDER-TT |
| 7 | ATR-Mako |
| 8 | WESROONA |
| 9 | AG-SPECTRUM |
| 10 | BC92157 |
| 11 | SKIPTON |
| 12 | CHARLTON |
| 13 | BC92156 |
| 14 | MARNOO |
| 15 | BLN3614 |
| 16 | AG-CASTLE |
| 17 | WESREO |
| 18 | MONTY |
| 19 | ATR-COBBLER |
| 20 | BC95042 |
| 21 | BLN3343-C00402P3 |
