# Supplementary material for: GWAS hints at pleiotropic roles for FLOWERING LOCUS T in flowering time and yield-related traits in canola
Source: BMC Genomics. 2019 Aug 6;20:636. doi: 10.1186/s12864-019-5964-y (PMC6685183; doi:10.1186/s12864-019-5964-y)
Supplement: Supplementary file 19 — Figure S1. Canola genotypes showing G X E interactions when grown under LD and SD conditions in controlled environment cabinet. Mean flowering time is estimated in days. Details of varieties shown here represented to BC accessions (Additional file 1: Table S1). (PPTX 213 kb) [file 12864_2019_5964_MOESM19_ESM.pptx]

## Slide 1
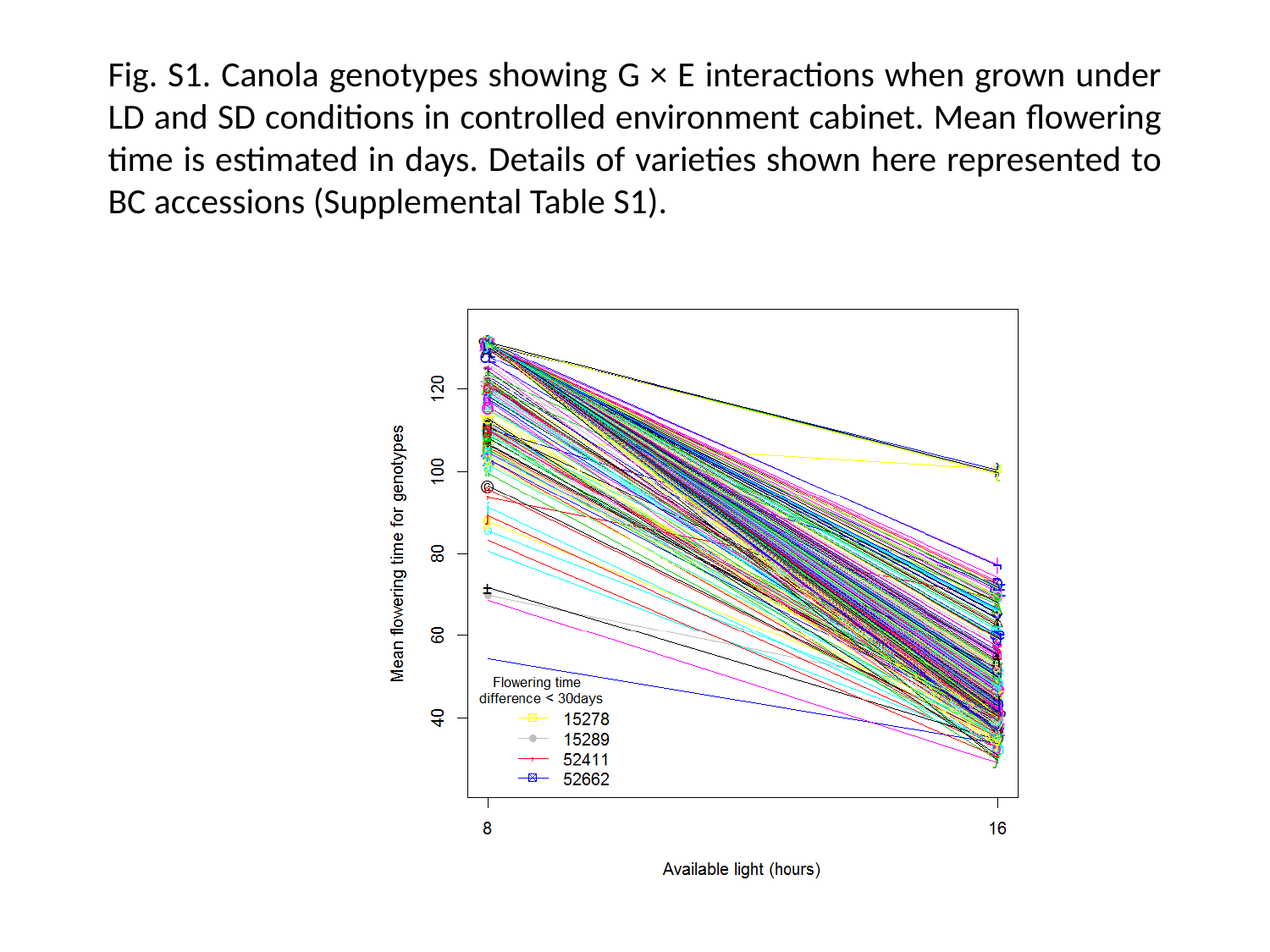

# Fig. S1. Canola genotypes showing G × E interactions when grown under LD and SD conditions in controlled environment cabinet. Mean flowering time is estimated in days. Details of varieties shown here represented to BC accessions (Supplemental Table S1).
