# Supplementary material for: GWAS hints at pleiotropic roles for FLOWERING LOCUS T in flowering time and yield-related traits in canola
Source: BMC Genomics. 2019 Aug 6;20:636. doi: 10.1186/s12864-019-5964-y (PMC6685183; doi:10.1186/s12864-019-5964-y)
Supplement: Supplementary file 20 — Figure S2. Genome-wide distribution (A) and density (B) of single nucleotide polymorphisms, in a genome wide association diversity panel of 368 Brassica napus accessions. Regions that are rich and poor SNP density are shown in dark and whitehorizontal bars, respectively. The number of SNP markers anchoring on different chromosomes (A1-A10 and C1-C9) of the physical map of the B.napus genome is given on the x-axis. (PPTX 959 kb) [file 12864_2019_5964_MOESM20_ESM.pptx]

## Slide 1
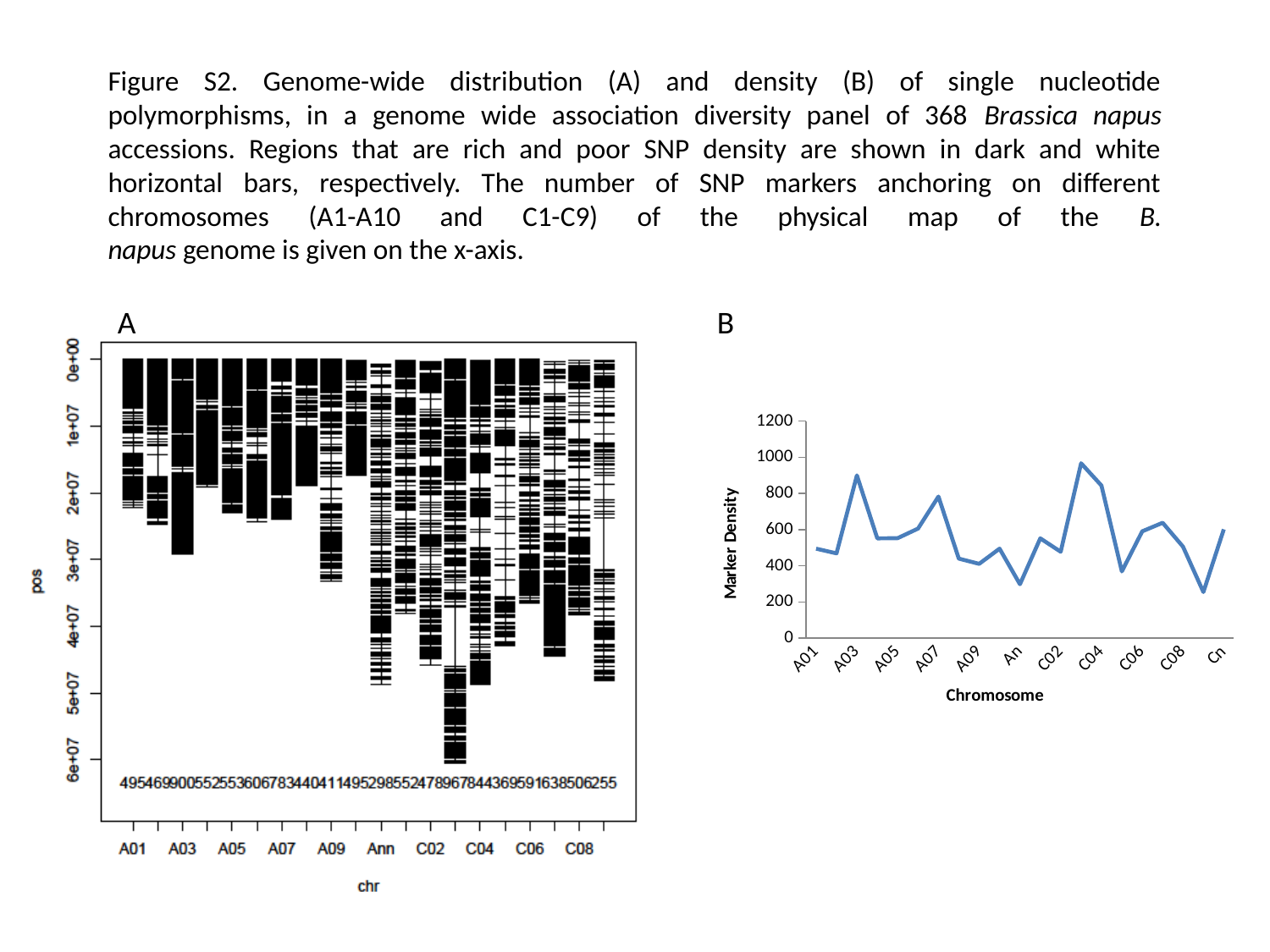

# Figure S2. Genome-wide distribution (A) and density (B) of single nucleotide polymorphisms, in a genome wide association diversity panel of 368 Brassica napus accessions. Regions that are rich and poor SNP density are shown in dark and white horizontal bars, respectively. The number of SNP markers anchoring on different chromosomes (A1-A10 and C1-C9) of the physical map of the B. napus genome is given on the x-axis.
A B
### Chart
| Category | |
|---|---|
| A01 | 495.0 |
| A02 | 469.0 |
| A03 | 900.0 |
| A04 | 552.0 |
| A05 | 553.0 |
| A06 | 606.0 |
| A07 | 783.0 |
| A08 | 440.0 |
| A09 | 411.0 |
| A10 | 495.0 |
| An | 298.0 |
| C01 | 552.0 |
| C02 | 478.0 |
| C03 | 967.0 |
| C04 | 844.0 |
| C05 | 369.0 |
| C06 | 591.0 |
| C07 | 638.0 |
| C08 | 506.0 |
| C09 | 255.0 |
| Cn | 602.0 |
