# Supplementary material for: GWAS hints at pleiotropic roles for FLOWERING LOCUS T in flowering time and yield-related traits in canola
Source: BMC Genomics. 2019 Aug 6;20:636. doi: 10.1186/s12864-019-5964-y (PMC6685183; doi:10.1186/s12864-019-5964-y)
Supplement: Supplementary file 21 — Figure S3. Genetic diversity and population structure in a GWAS panel of 368 Brassica napus accessions. Three clusters designated as I, II and III represent predominantly Chinese, European, and Australian accessions, respectively. Details of accessions are given in Additional file 1: Table S1. (PPTX 1670 kb) [file 12864_2019_5964_MOESM21_ESM.pptx]

## Slide 1
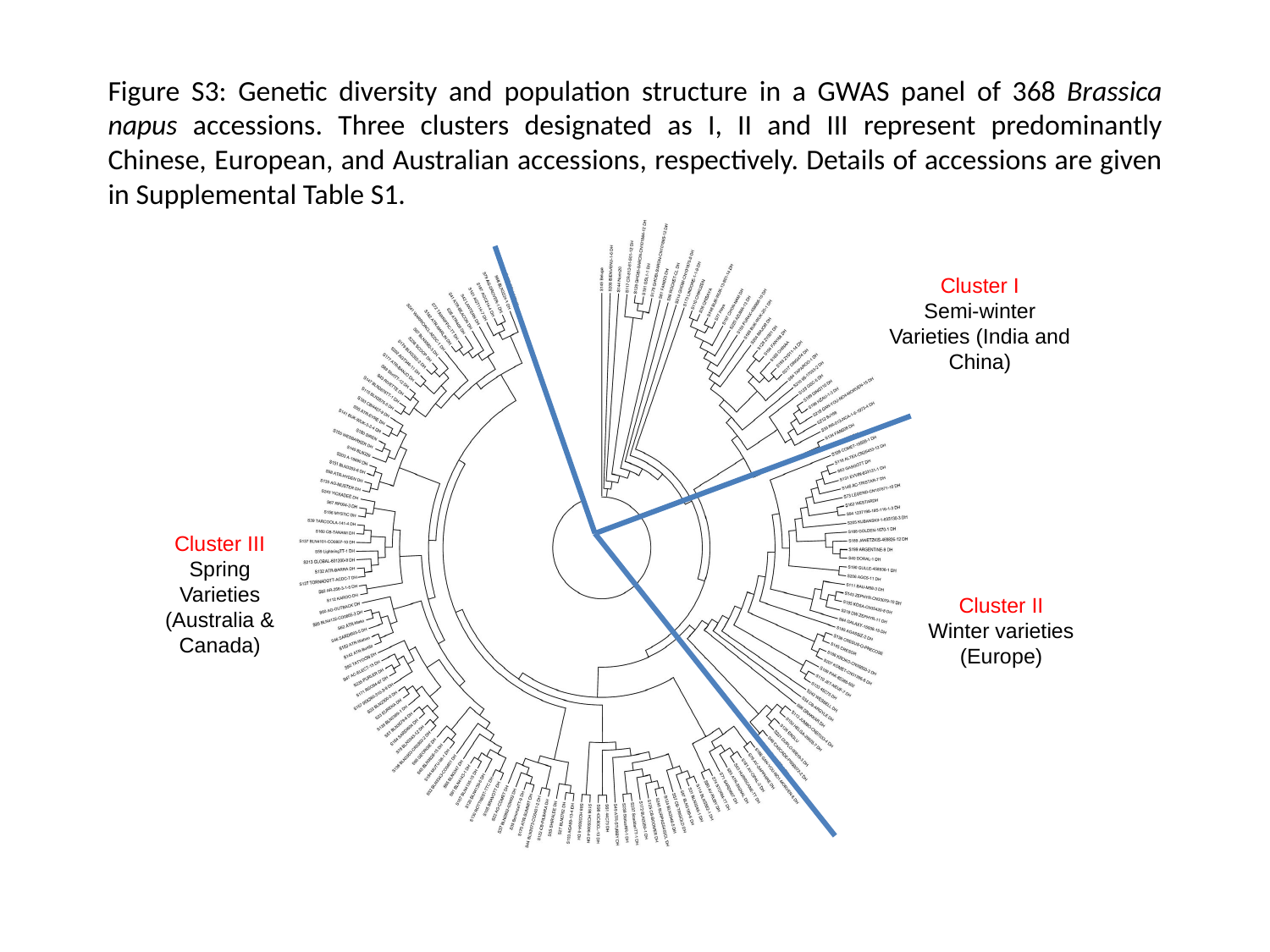

# Figure S3: Genetic diversity and population structure in a GWAS panel of 368 Brassica napus accessions. Three clusters designated as I, II and III represent predominantly Chinese, European, and Australian accessions, respectively. Details of accessions are given in Supplemental Table S1.
Cluster I
Semi-winter
Varieties (India and China)
Cluster III
Spring
Varieties
(Australia & Canada)
Cluster II
Winter varieties
(Europe)
