# Supplementary material for: GWAS hints at pleiotropic roles for FLOWERING LOCUS T in flowering time and yield-related traits in canola
Source: BMC Genomics. 2019 Aug 6;20:636. doi: 10.1186/s12864-019-5964-y (PMC6685183; doi:10.1186/s12864-019-5964-y)
Supplement: Supplementary file 22 — Figure S4. Principal components (PC1 and PC2) analysis showing population structure in a GWAS diversity panel of 368 B. napus accessions. Three major clusters designated as I, II, and III, consistent with the cluster analysis (Additional file 20: Figure S2). (PPTX 2450 kb) [file 12864_2019_5964_MOESM22_ESM.pptx]

## Slide 1
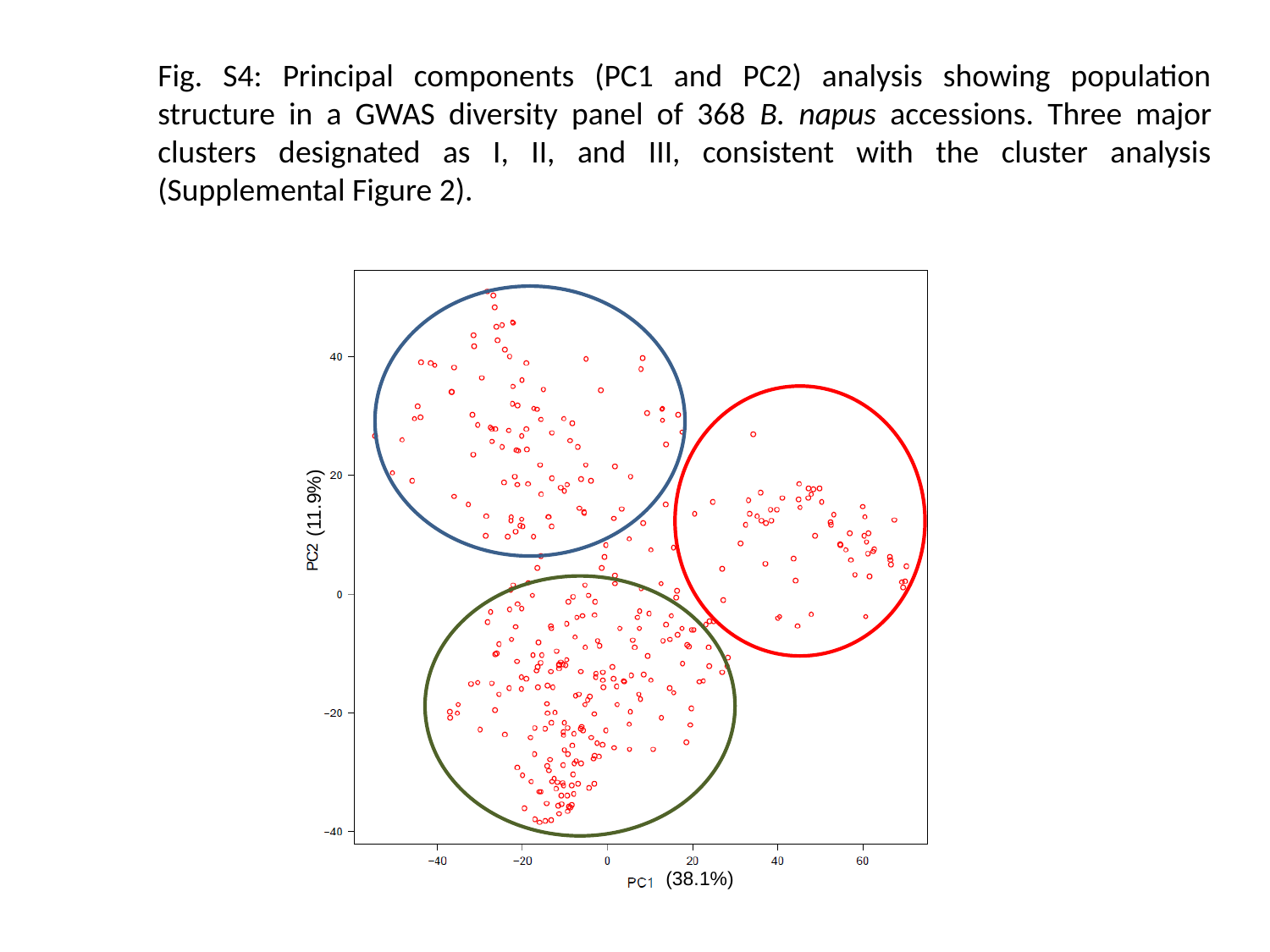

# Fig. S4: Principal components (PC1 and PC2) analysis showing population structure in a GWAS diversity panel of 368 B. napus accessions. Three major clusters designated as I, II, and III, consistent with the cluster analysis (Supplemental Figure 2).
(11.9%)
(38.1%)
