# Supplementary material for: GWAS hints at pleiotropic roles for FLOWERING LOCUS T in flowering time and yield-related traits in canola
Source: BMC Genomics. 2019 Aug 6;20:636. doi: 10.1186/s12864-019-5964-y (PMC6685183; doi:10.1186/s12864-019-5964-y)
Supplement: Supplementary file 23 — Figure S5. The average linkage disequilibrium (LD) decays (r2) approach 0.02 when distance between SNPs was approximately 200 Kb. Distance in bp is shown on X-axis. (PPTX 135 kb) [file 12864_2019_5964_MOESM23_ESM.pptx]

## Slide 1
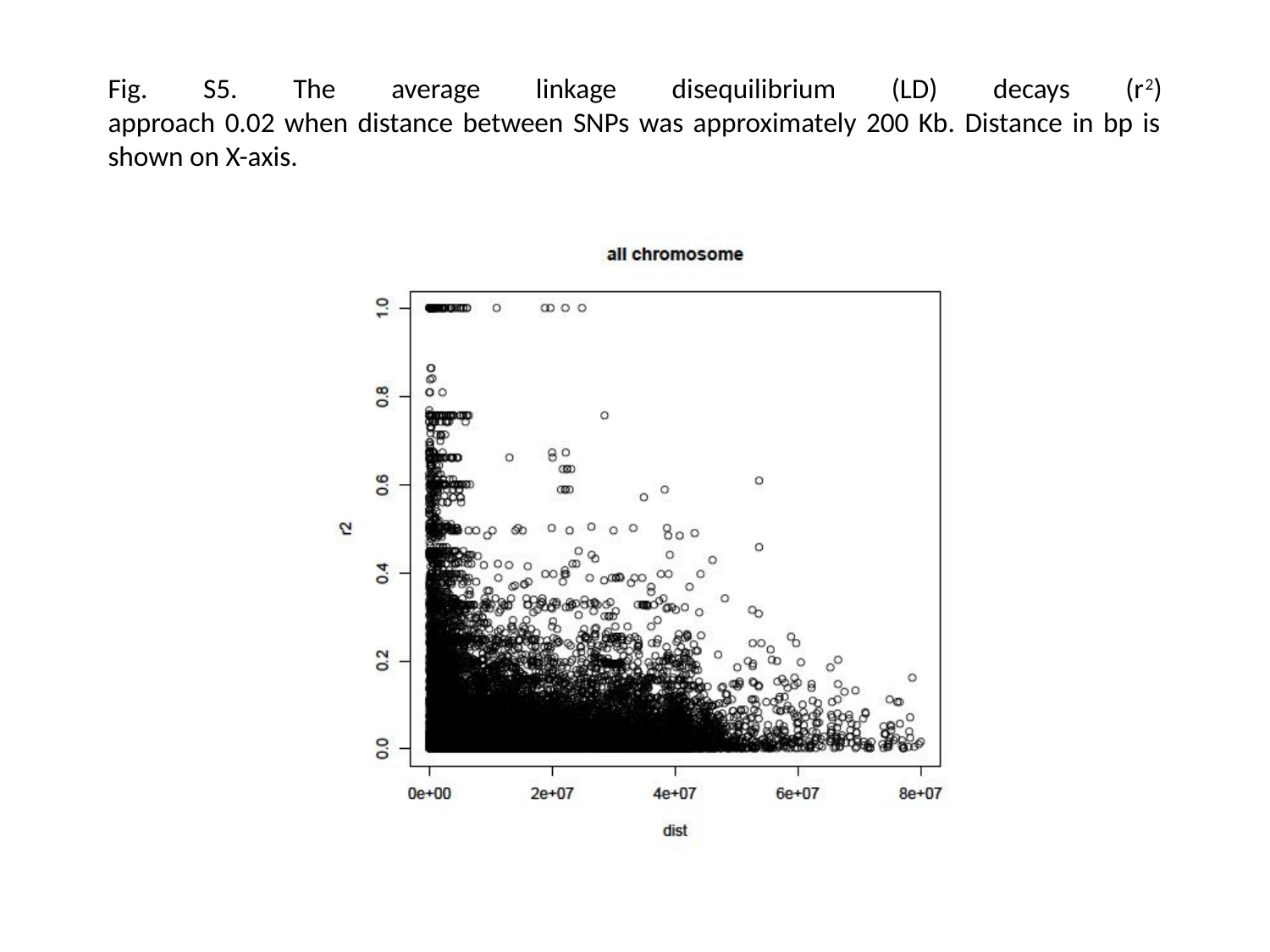

# Fig. S5. The average linkage disequilibrium (LD) decays (r2) approach 0.02 when distance between SNPs was approximately 200 Kb. Distance in bp is shown on X-axis.
