# Supplementary material for: GWAS hints at pleiotropic roles for FLOWERING LOCUS T in flowering time and yield-related traits in canola
Source: BMC Genomics. 2019 Aug 6;20:636. doi: 10.1186/s12864-019-5964-y (PMC6685183; doi:10.1186/s12864-019-5964-y)
Supplement: Supplementary file 24 — Figure S6. Candidate genes located within 200 kb from the significant SNPs associated with flowering time in a GWAS panel of canola. Accessions were grown under long day (LD, 14 h light), short day (SD, 8 h light) treatments in controlled environments (CE) and three field conditions at Wagga Wagga [in single rows: WAG-FT (Row) and plots: WAG-FT (Plots)] and Condobolin [in plots: CON-FT (Plots). Response to photoperiod was estimated as the difference between LD and SD treatments (days). Details are given in Additional file 11: Table S11. (PPTX 189 kb) [file 12864_2019_5964_MOESM24_ESM.pptx]

## Slide 1
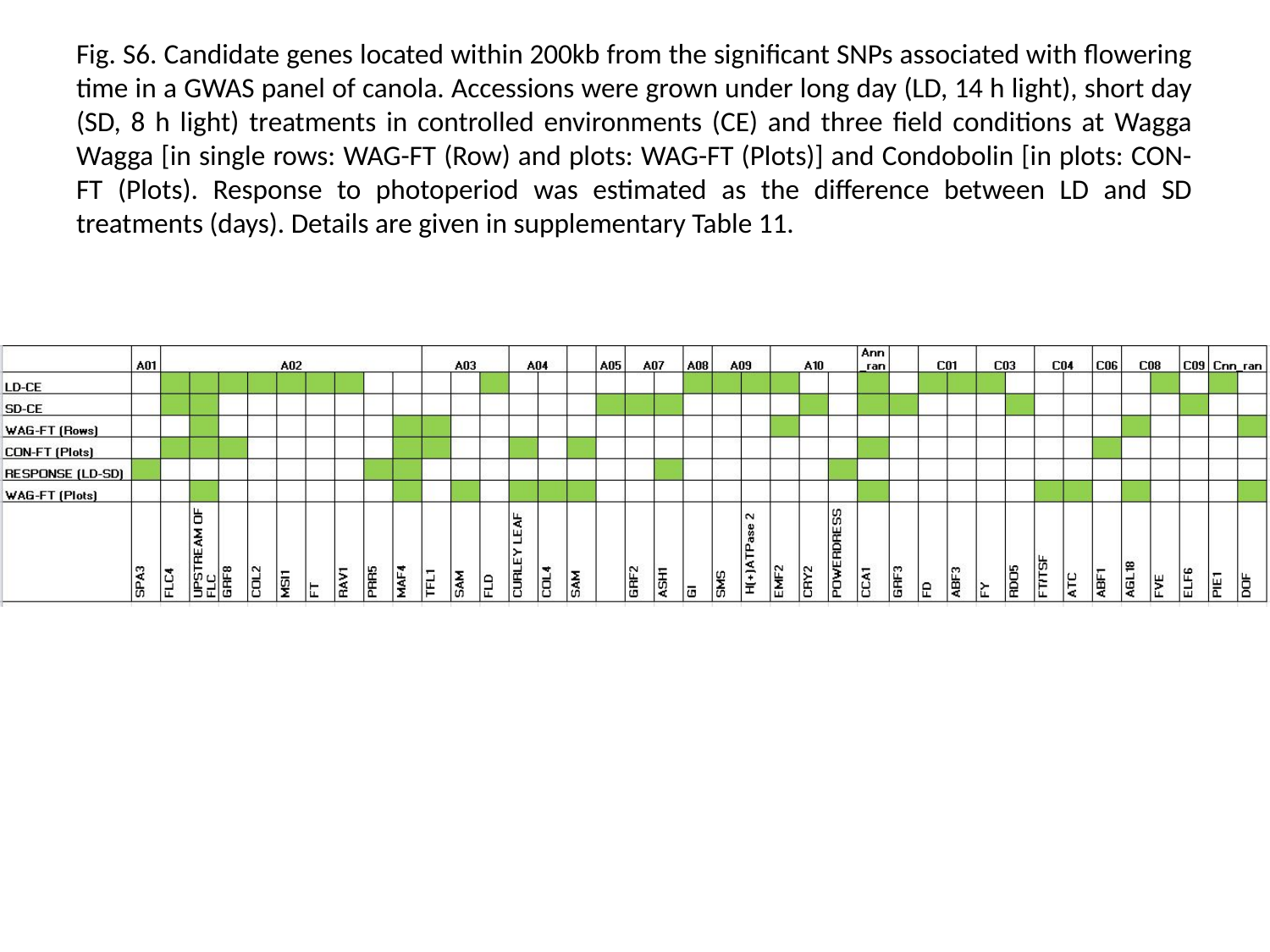

# Fig. S6. Candidate genes located within 200kb from the significant SNPs associated with flowering time in a GWAS panel of canola. Accessions were grown under long day (LD, 14 h light), short day (SD, 8 h light) treatments in controlled environments (CE) and three field conditions at Wagga Wagga [in single rows: WAG-FT (Row) and plots: WAG-FT (Plots)] and Condobolin [in plots: CON-FT (Plots). Response to photoperiod was estimated as the difference between LD and SD treatments (days). Details are given in supplementary Table 11.
