# Supplementary material for: GWAS hints at pleiotropic roles for FLOWERING LOCUS T in flowering time and yield-related traits in canola
Source: BMC Genomics. 2019 Aug 6;20:636. doi: 10.1186/s12864-019-5964-y (PMC6685183; doi:10.1186/s12864-019-5964-y)
Supplement: Supplementary file 25 — Figure S7. (A). Frequency distribution of shoot biomass in a SAgS DH population phenotyped across 2014–2016 growing environments. (B). Frequency distribution of fractional ground cover, measured as NSVI with a hand-held GreenSeeker machine, in a SAgS DH population phenotyped across 2015–2016 growing environments). (C). Frequency distribution of days to flower in a SAgS DH population phenotyped across four environments (2013–2016). Phenotypic data of 2013 and 2014 was published previously (Raman et al. 2016 [12, 13, 52]). (D). Frequency distribution of plant height and plant emergence in a SAgS DH population phenotyped in 2016 growing environments. (E). Frequency distribution of grain yield in a SAgS DH population phenotyped across four environments (2013–2016). Phenotypic data of 2013 and 2014 experiments was published previously (Raman et al. 2016 [12, 13, 35]). (PPTX 5970 kb) [file 12864_2019_5964_MOESM25_ESM.pptx]

## Slide 1
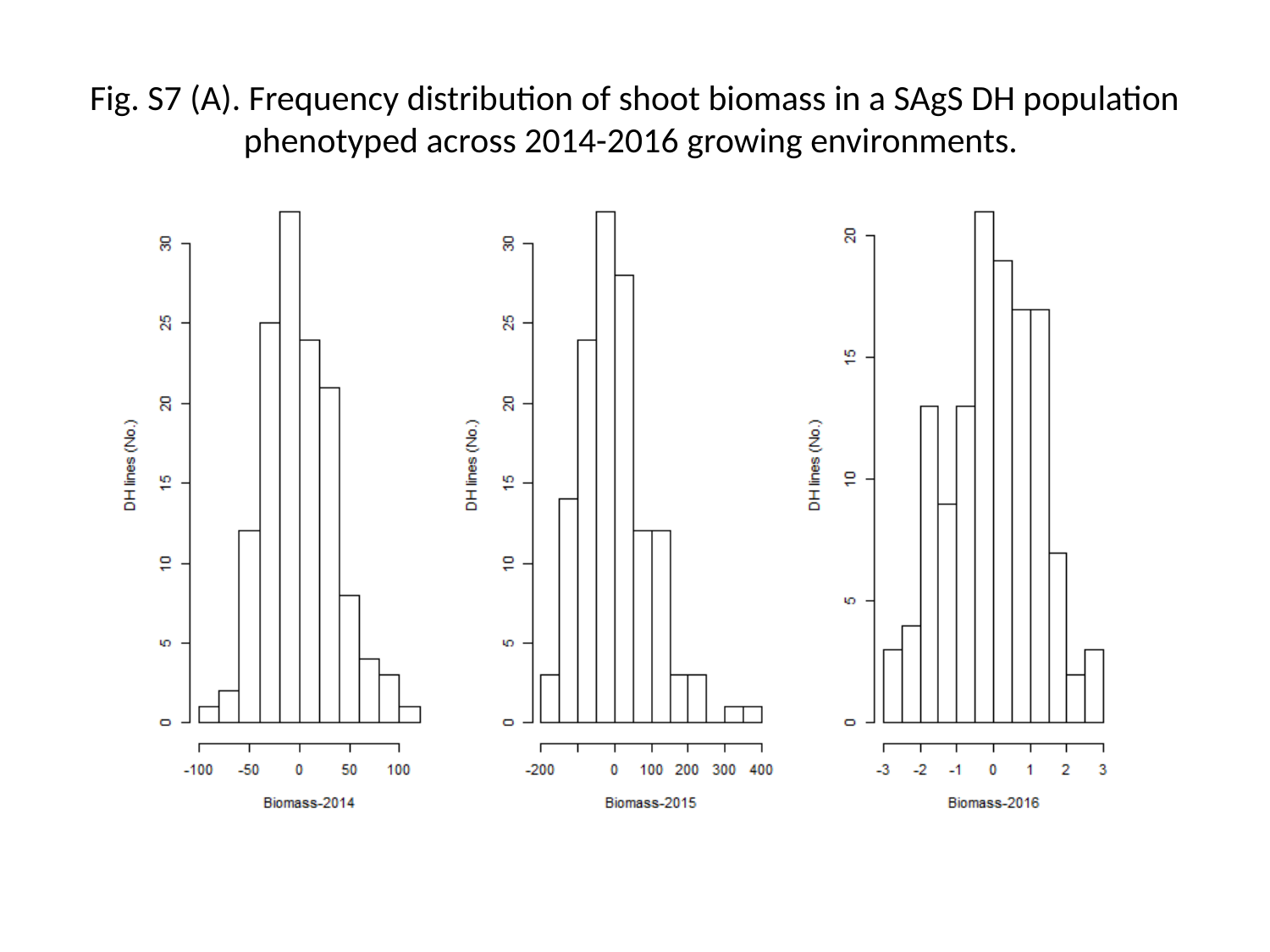

# Fig. S7 (A). Frequency distribution of shoot biomass in a SAgS DH population phenotyped across 2014-2016 growing environments.

## Slide 2
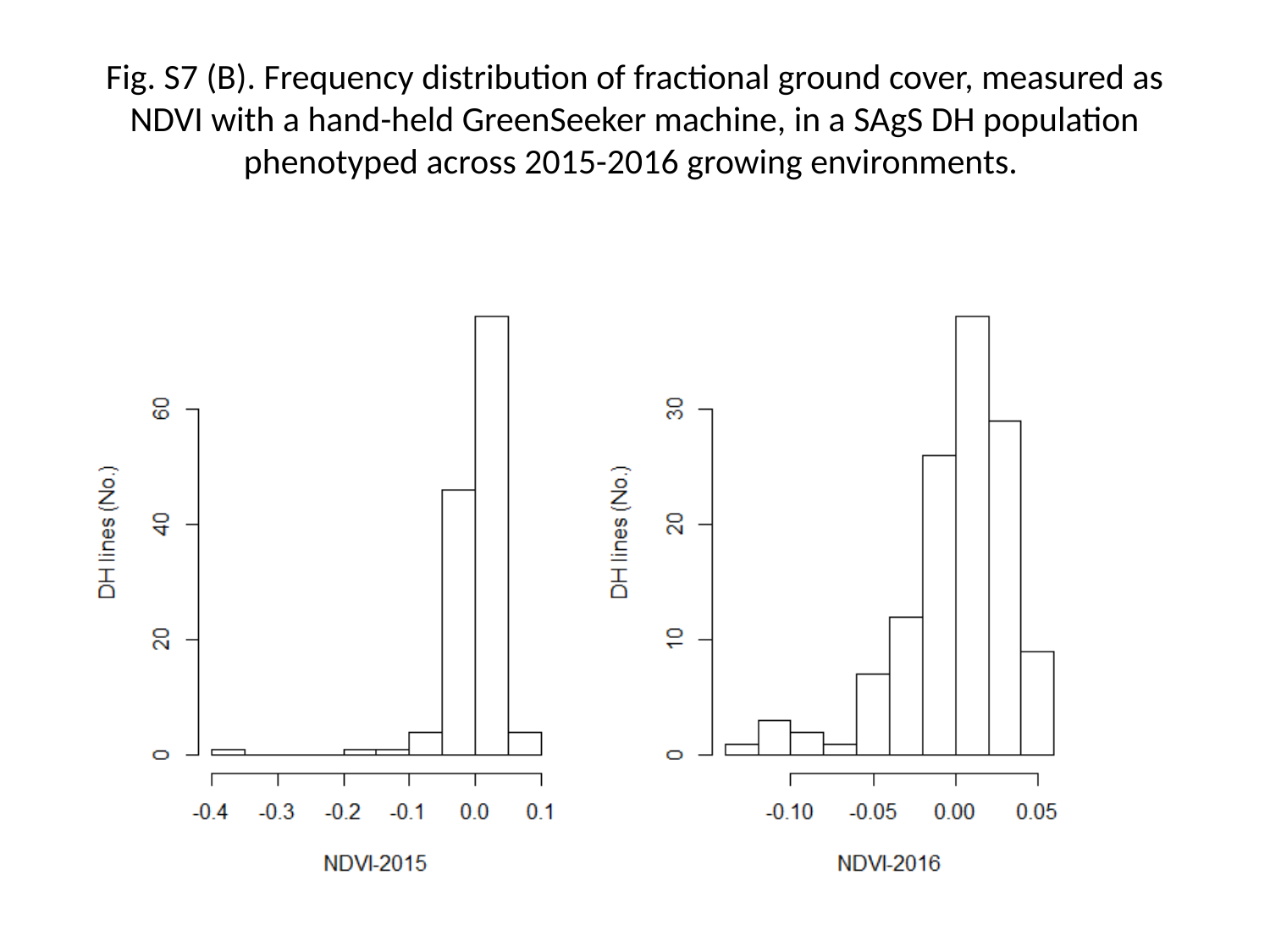

# Fig. S7 (B). Frequency distribution of fractional ground cover, measured as NDVI with a hand-held GreenSeeker machine, in a SAgS DH population phenotyped across 2015-2016 growing environments.

## Slide 3
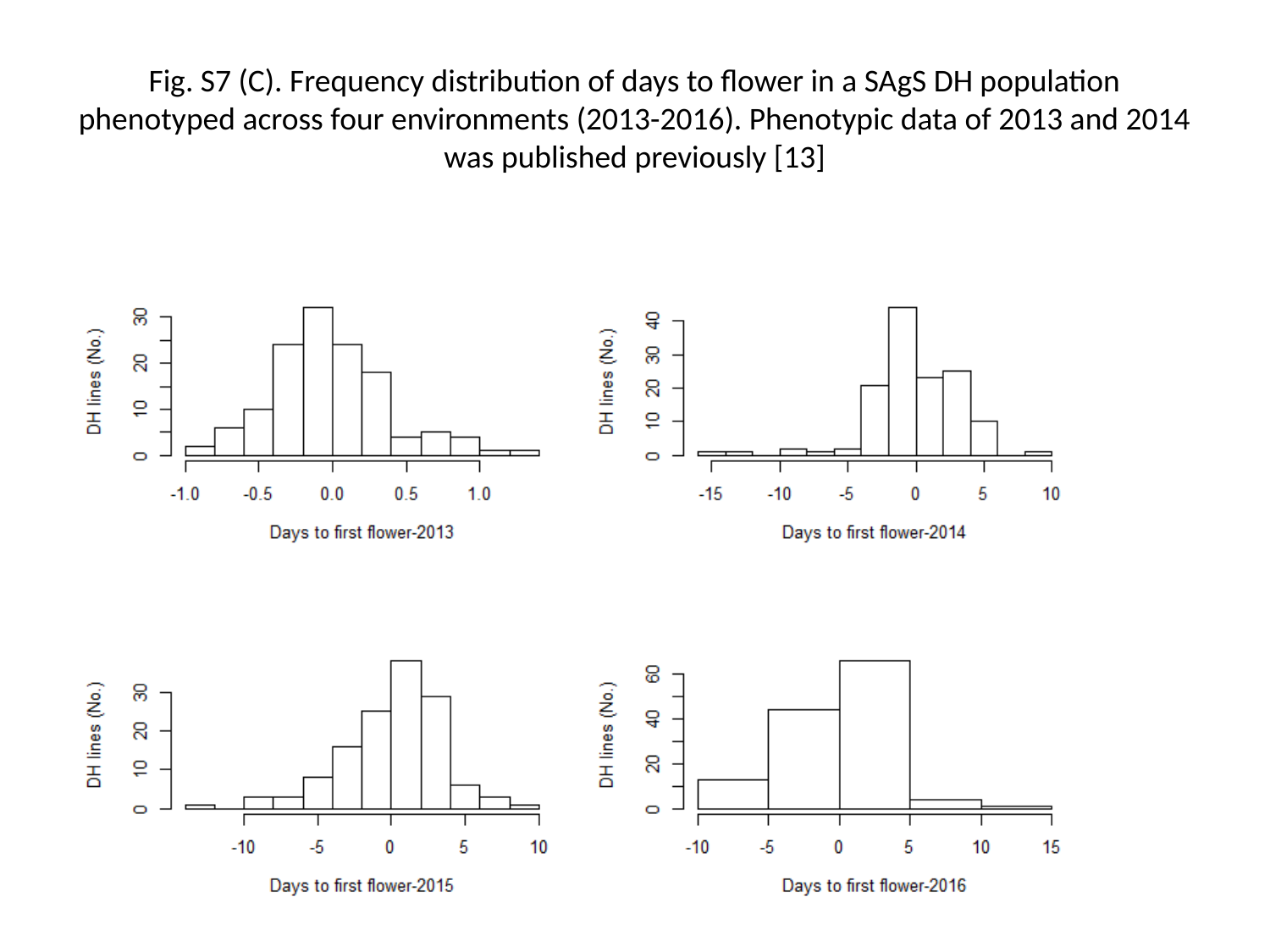

# Fig. S7 (C). Frequency distribution of days to flower in a SAgS DH population phenotyped across four environments (2013-2016). Phenotypic data of 2013 and 2014 was published previously [13]

## Slide 4
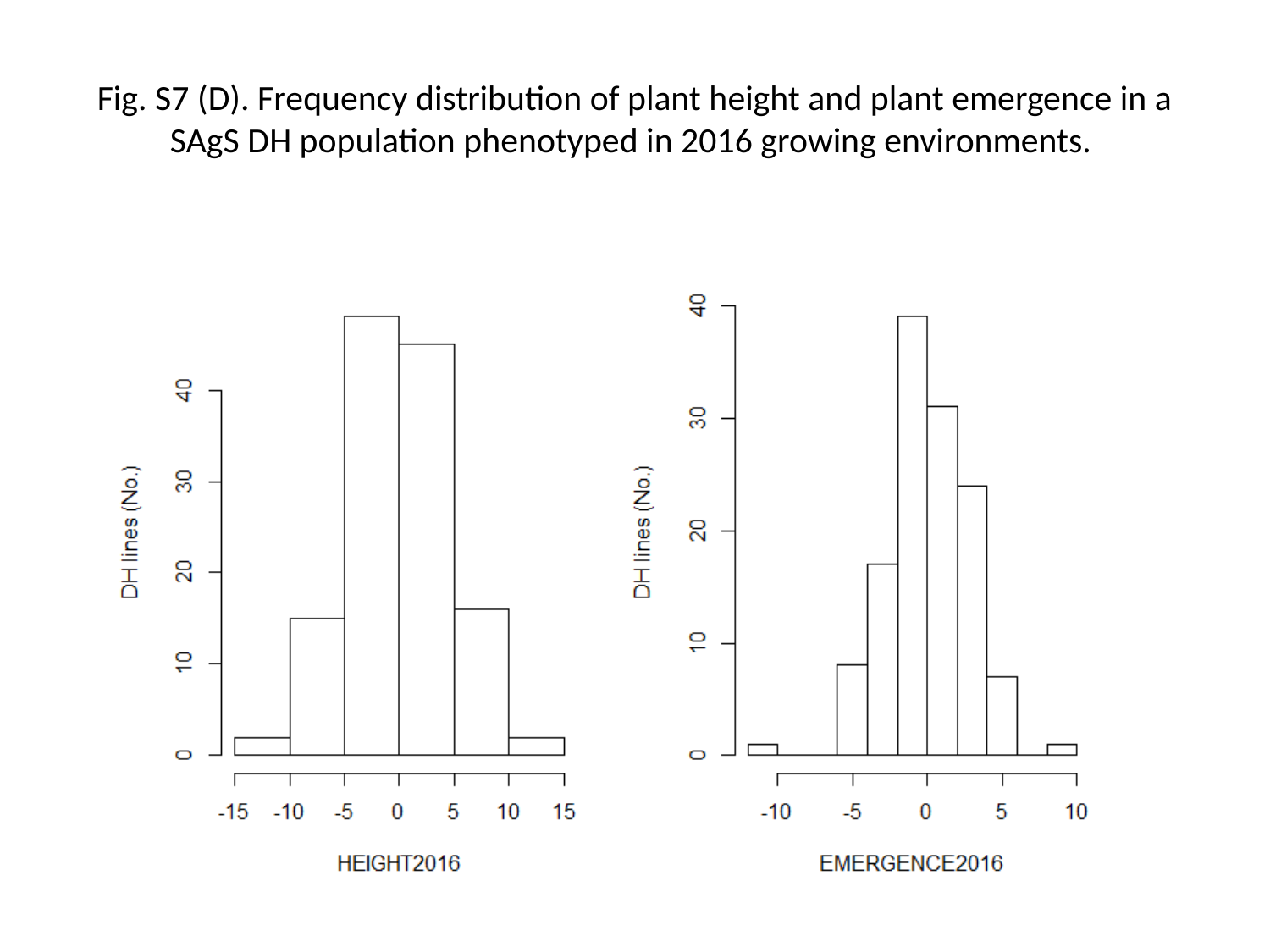

# Fig. S7 (D). Frequency distribution of plant height and plant emergence in a SAgS DH population phenotyped in 2016 growing environments.

## Slide 5
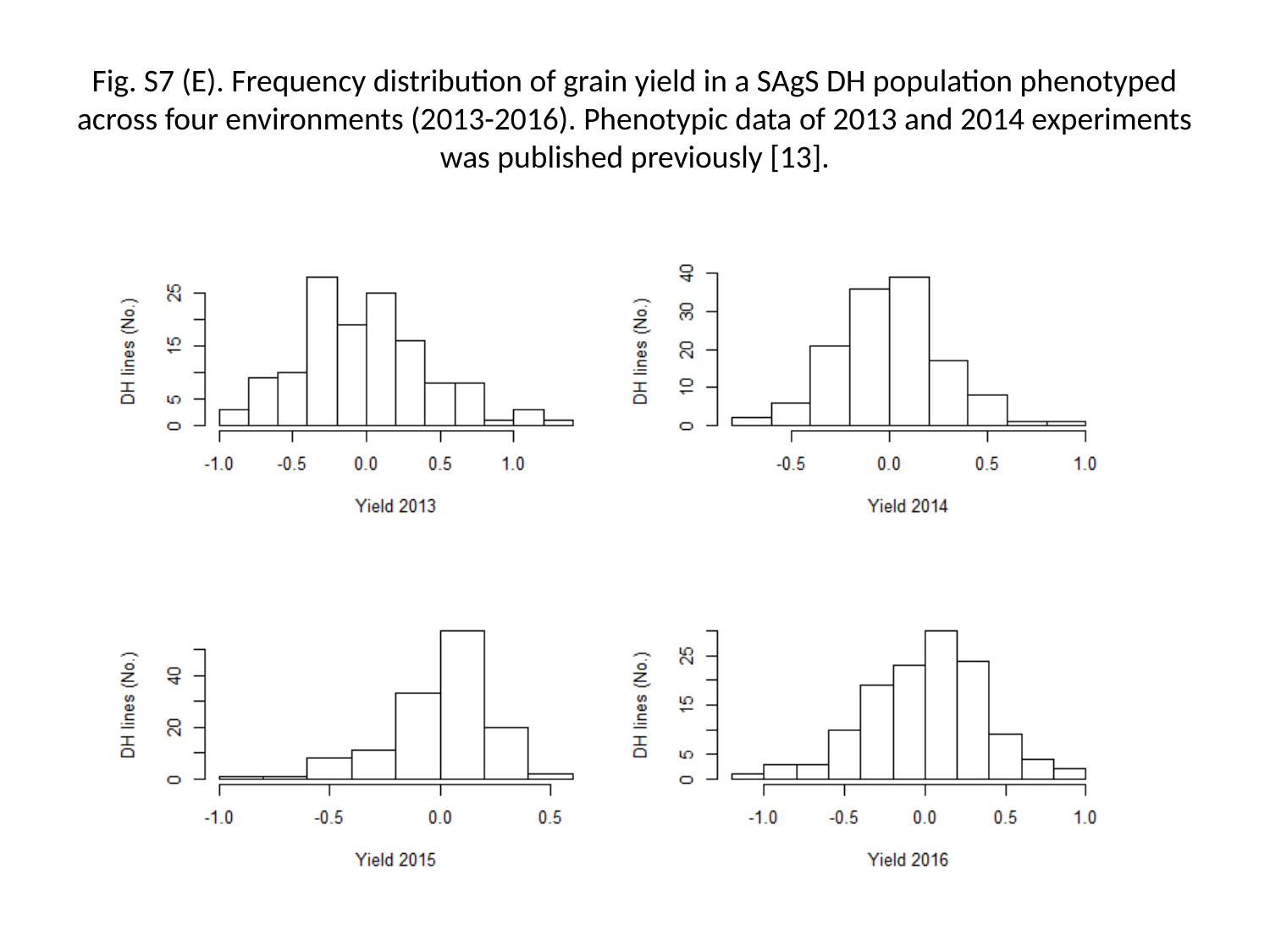

# Fig. S7 (E). Frequency distribution of grain yield in a SAgS DH population phenotyped across four environments (2013-2016). Phenotypic data of 2013 and 2014 experiments was published previously [13].
