# Supplementary material for: GWAS hints at pleiotropic roles for FLOWERING LOCUS T in flowering time and yield-related traits in canola
Source: BMC Genomics. 2019 Aug 6;20:636. doi: 10.1186/s12864-019-5964-y (PMC6685183; doi:10.1186/s12864-019-5964-y)
Supplement: Supplementary file 26 — Figure S8. A: Regions of homology between the B. napus FT regions and block C from A. thaliana. Putative binding sites are indicated based on ref . BN_chrC06 is upstream from BnaC06g27090D, BN_chrA07 is upstream from BnaA07g25310D, and BN_chrA02 is upstream from BnaA02g12130D. A corresponding block C region for BnaC02g45250D could not be identified. B: Regions of homology between the B. napus FT regions and block E from A. thaliana. Putative binding sites are indicated based on ref. . BN_chrA07 is downstream from BnaA07g25310D, BN_chrC02rnd is downstream from BnaC02g45250D, BN_chrA02 is downstream from BnaA02g12130D and BN_chrC06 is downstream from BnaC06g27090D. C: Summary of SNP and Indel variation in the B. napus FT gene BnaA02g12130D across 21 lines. The gene model is shown below the plot. Key: Insertions = triangle, deletions = inverted triangle, SNPs = dots, red = nonsynonymous change. D: Summary of SNP and Indel variation in the B. napus FT gene BnaA07g25310D across 21 lines. The gene model is shown below the plot. Key: Insertions = triangle, deletions = inverted triangle, SNPs = dots, red = nonsynonymous change. E: Summary of SNP and Indel variation in the B. napus FT gene BnaC02g45250D across 21 lines. The gene model is shown below the plot. Key: Insertions = triangle, deletions = inverted triangle, SNPs = dots, red = nonsynonymous change. F. Summary of SNP and Indel variation in the B. napus FT gene BnaC06g27090D across 21 lines (only a subset of lines are shown). The gene model is shown below the plot. Key: Insertions = triangle, deletions = inverted triangle, SNPs = dots, red = nonsynonymous change. (PPTX 3860 kb) [file 12864_2019_5964_MOESM26_ESM.pptx]

## Slide 1
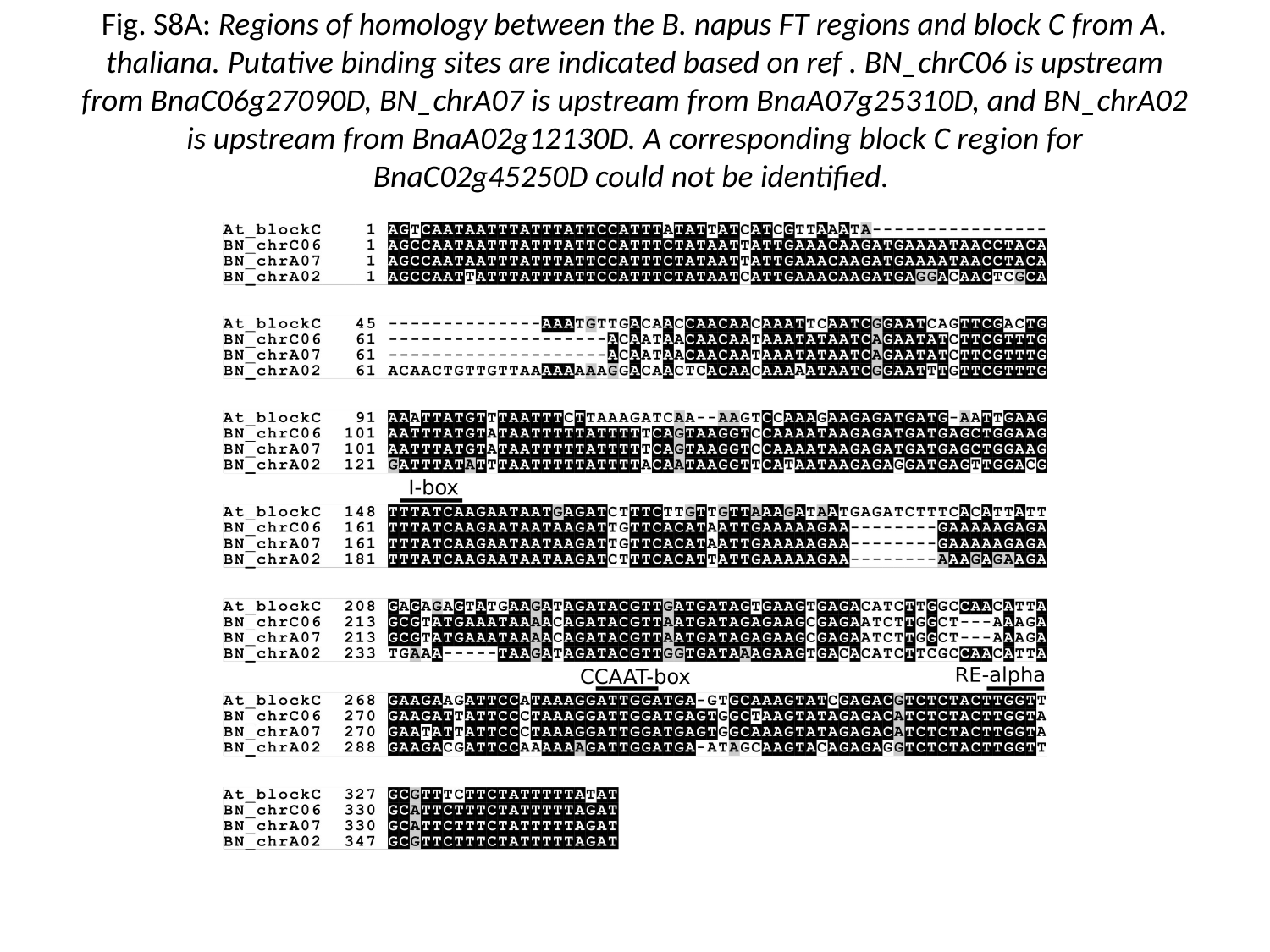

# Fig. S8A: Regions of homology between the B. napus FT regions and block C from A. thaliana. Putative binding sites are indicated based on ref . BN_chrC06 is upstream from BnaC06g27090D, BN_chrA07 is upstream from BnaA07g25310D, and BN_chrA02 is upstream from BnaA02g12130D. A corresponding block C region for BnaC02g45250D could not be identified.

## Slide 2
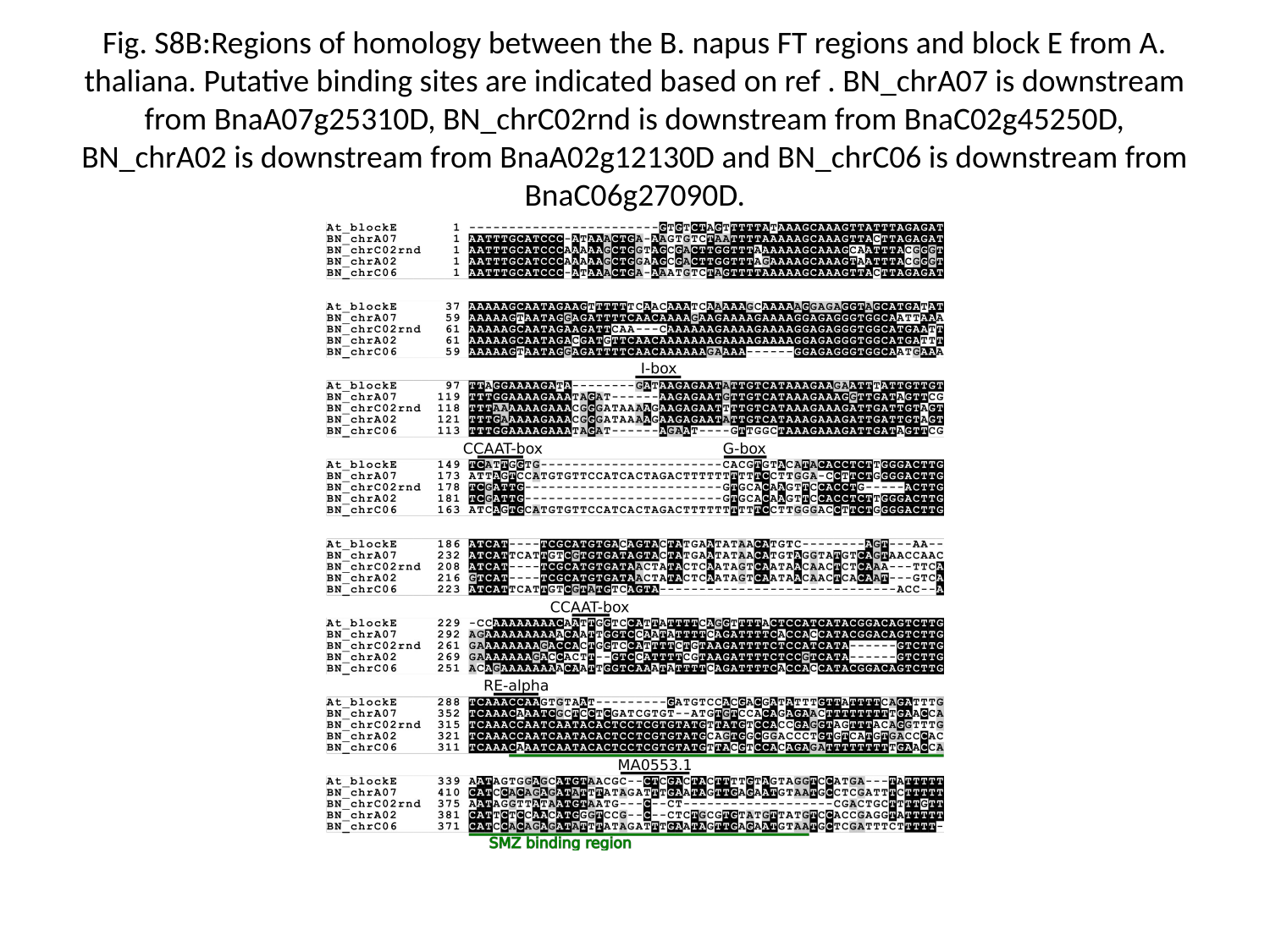

# Fig. S8B:Regions of homology between the B. napus FT regions and block E from A. thaliana. Putative binding sites are indicated based on ref . BN_chrA07 is downstream from BnaA07g25310D, BN_chrC02rnd is downstream from BnaC02g45250D, BN_chrA02 is downstream from BnaA02g12130D and BN_chrC06 is downstream from BnaC06g27090D.

## Slide 3
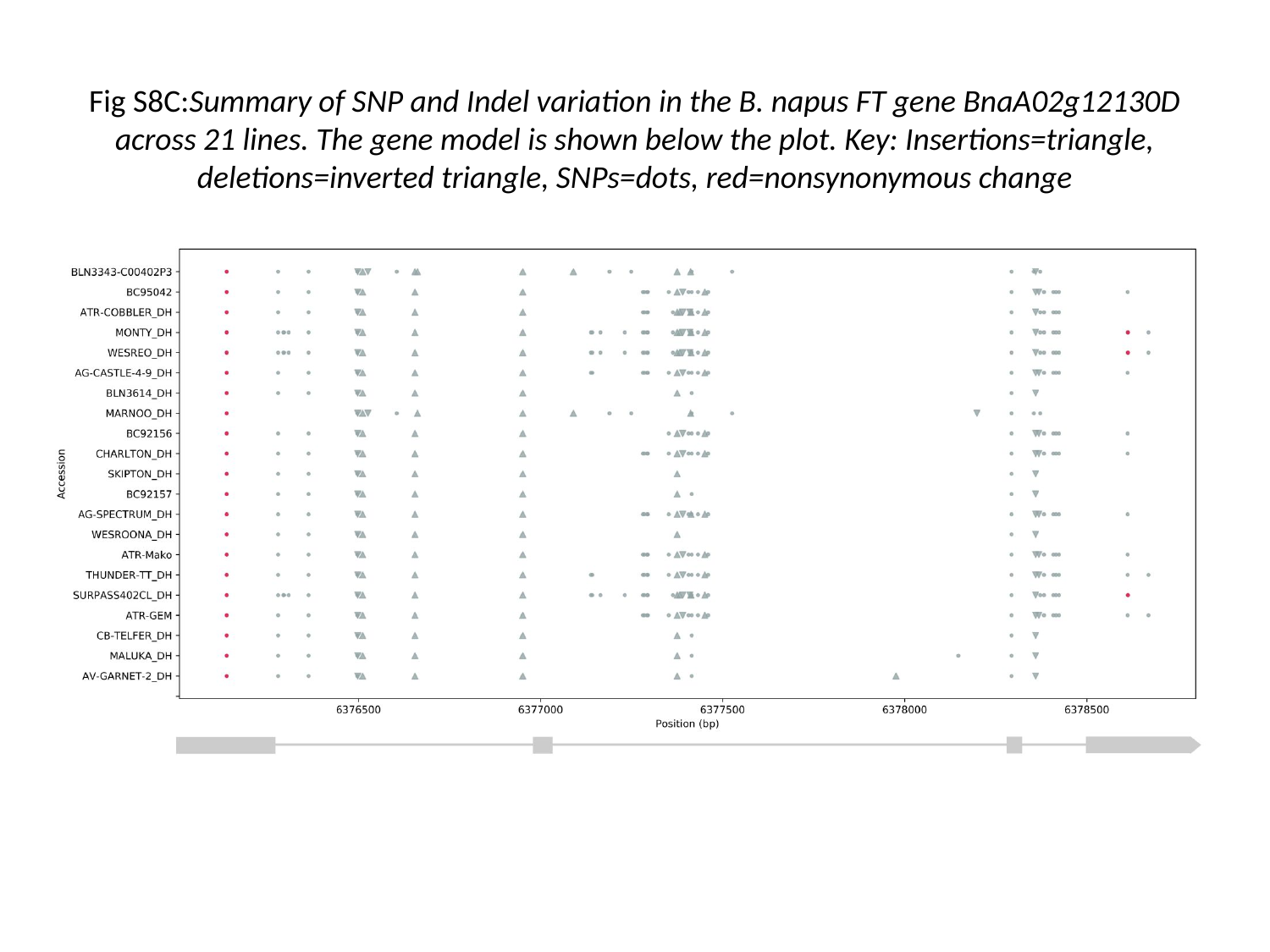

# Fig S8C:Summary of SNP and Indel variation in the B. napus FT gene BnaA02g12130D across 21 lines. The gene model is shown below the plot. Key: Insertions=triangle, deletions=inverted triangle, SNPs=dots, red=nonsynonymous change

## Slide 4
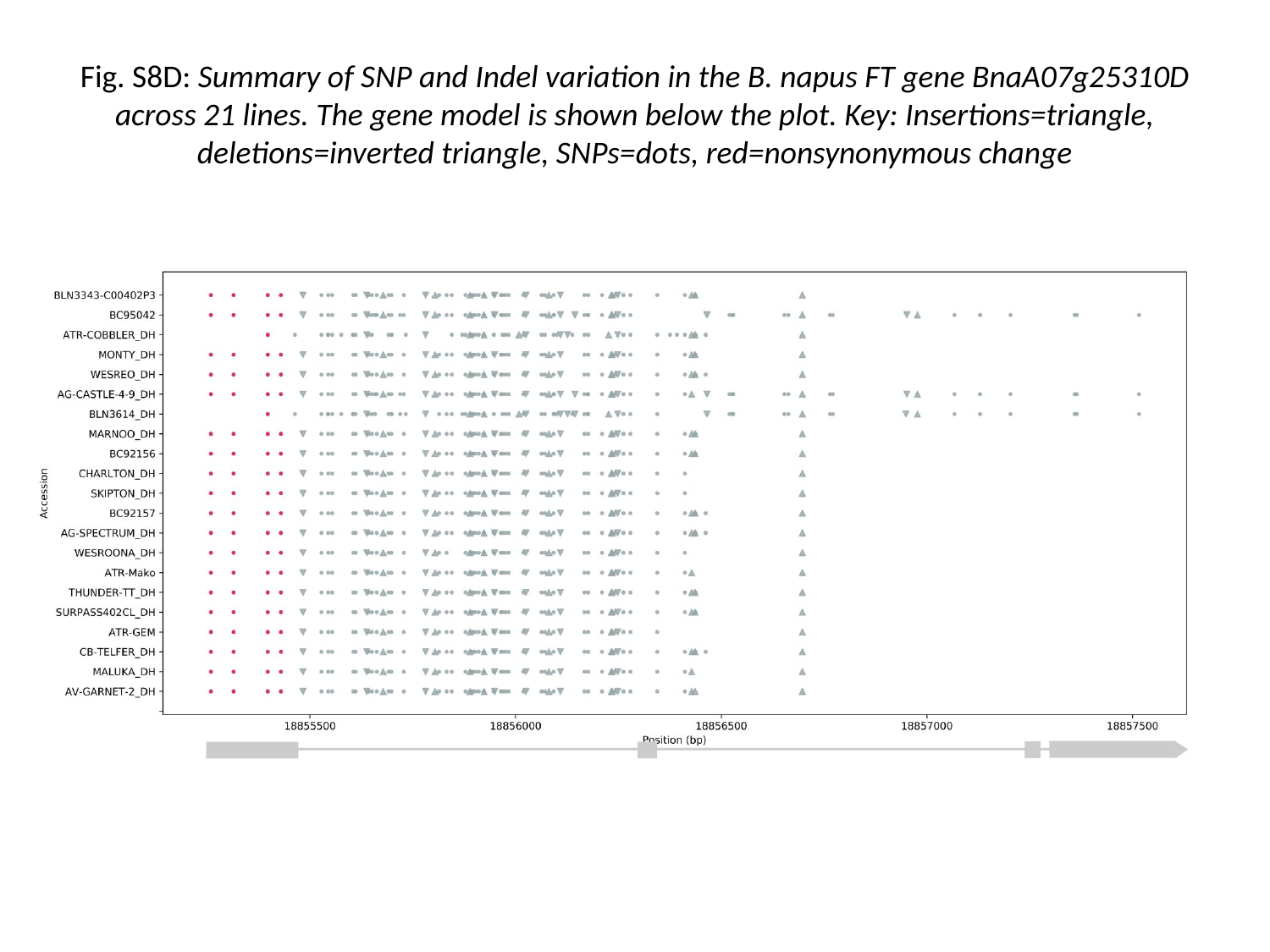

# Fig. S8D: Summary of SNP and Indel variation in the B. napus FT gene BnaA07g25310D across 21 lines. The gene model is shown below the plot. Key: Insertions=triangle, deletions=inverted triangle, SNPs=dots, red=nonsynonymous change

## Slide 5
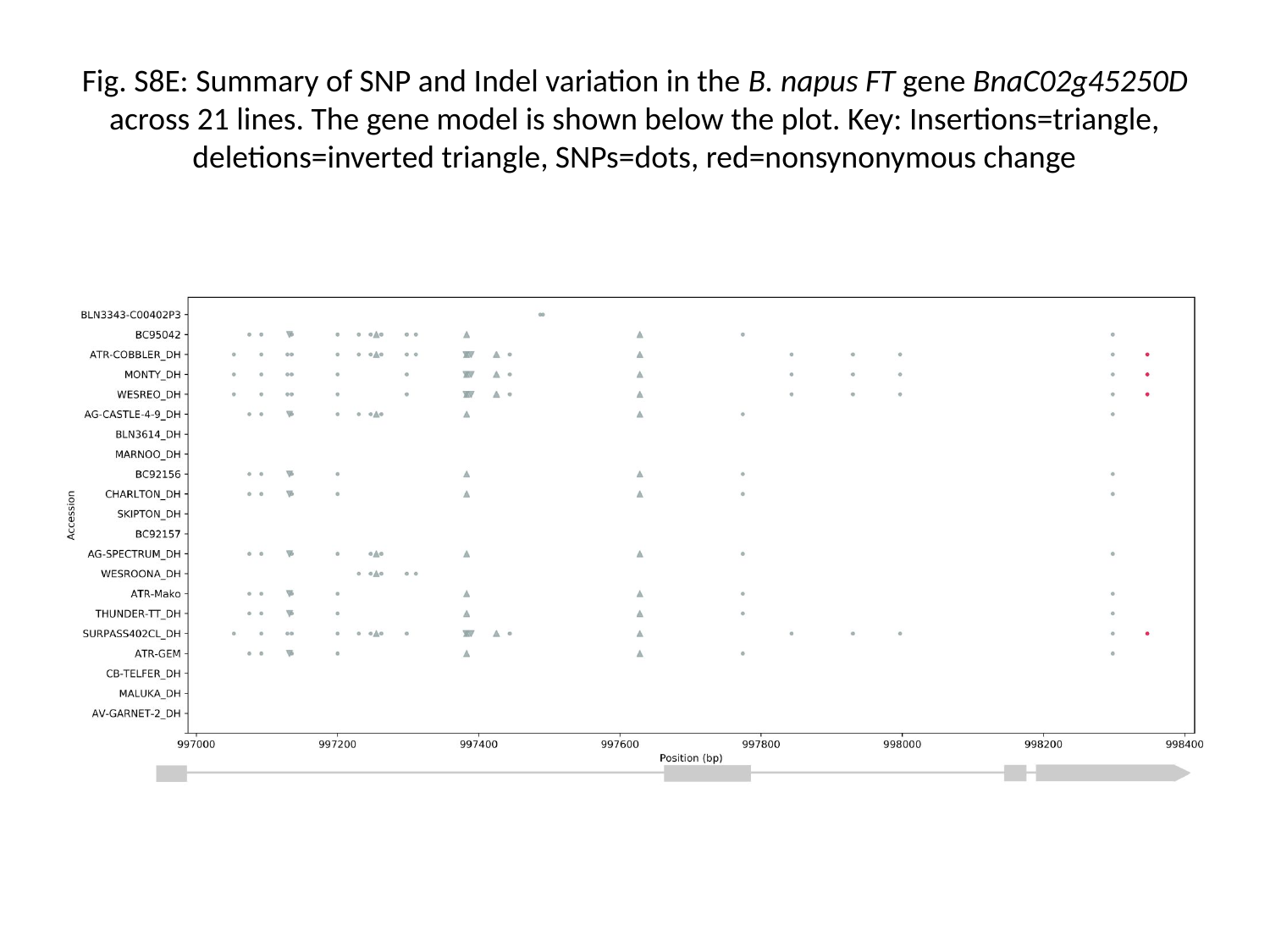

# Fig. S8E: Summary of SNP and Indel variation in the B. napus FT gene BnaC02g45250D across 21 lines. The gene model is shown below the plot. Key: Insertions=triangle, deletions=inverted triangle, SNPs=dots, red=nonsynonymous change

## Slide 6
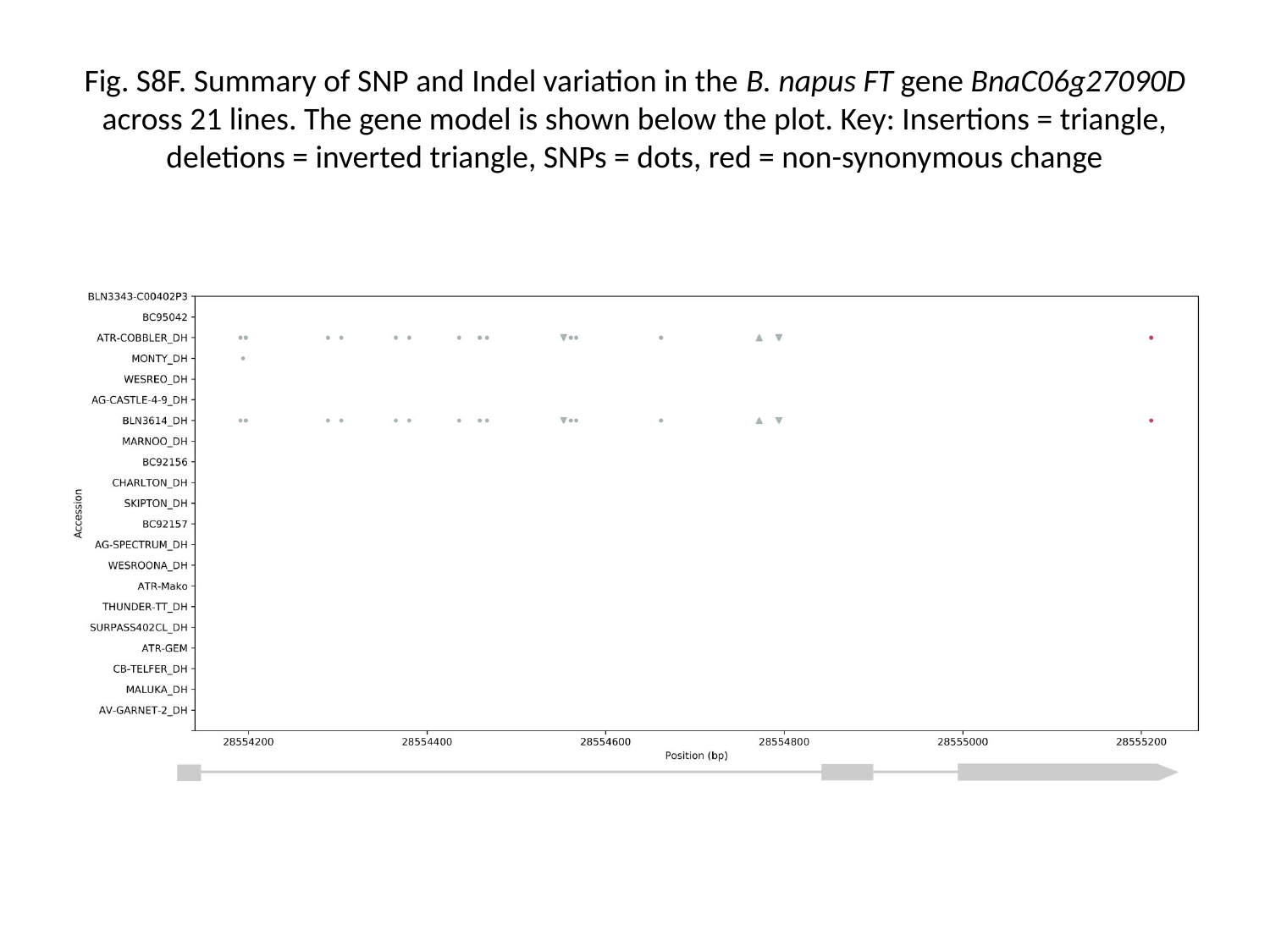

# Fig. S8F. Summary of SNP and Indel variation in the B. napus FT gene BnaC06g27090D across 21 lines. The gene model is shown below the plot. Key: Insertions = triangle, deletions = inverted triangle, SNPs = dots, red = non-synonymous change
